# Evaluating Missing Data Imputation Strategies for Environmental Mixture Models: A Simulation Study and Applied Analysis

**DOI:** 10.1080/26941899.2026.2696661

**Published:** 2026-07-14

**Authors:** Yvonne S. Boafo, Sayed Mostafa, Emmanuel Obeng-Gyasi

**Affiliations:** a Department of Built Environment, North Carolina Agricultural and Technical State University, Greensboro, NC, USA; b Department of Mathematics and Statistics, North Carolina Agricultural and Technical State University, Greensboro, NC, USA

**Keywords:** Environmental mixtures, multiple imputation, Bayesian kernel machine regression (BKMR), quantile g-computation (Q-gcomp), weighted quantile sum (WQS) regression

## Abstract

Missing data are common in environmental mixture studies and can bias inference if not properly addressed. This study evaluates how different imputation strategies influence variable selection performance in six mixture modeling frameworks: Weighted Quantile Sum (WQS), Bayesian WQS (BWQS), Quantile g-computation (Q-gcomp), Bayesian Kernel Machine Regression (BKMR), Elastic Net, and Least Absolute Shrinkage and Selection Operator (LASSO). Using Monte Carlo simulations (MCS), we generated multivariate normal (MVNORM) and multivariate t- (MVT) distributed exposures under linear and nonlinear outcome structures. Missingness was introduced at 25% for exposure and outcome variables, and at 5%–25% under both missing-at-random (MAR) and missing-not-at-random (MNAR) mechanisms. The study compares single imputation (SI) methods (mean, median, and k-nearest neighbors [KNN]) with multiple imputation approaches [MI] (MICE and Amelia) based on sensitivity (SE), specificity (SP), and false discovery rate (FDR). Across simulations, MI consistently improved variable selection performance under MAR, whereas SI and listwise deletion increased variability and reduced accuracy. Under MNAR, performance declined across all methods, with greater instability observed in flexible models such as BKMR and WQS. Q-gcomp demonstrated the most consistent balance across SE, SP, and FDR and remained relatively robust to violations of the MAR assumption. Additional analyses highlight the importance of imputation model specification: excluding relevant covariates (e.g. income) from the imputation process increased variability and reduced stability, particularly for flexible models and binary outcomes. Application to NHANES 2007–2014 data (*n* = 8,233) showed that MI improved the stability of variable importance estimates, with BWQS and Q-gcomp yielding more reproducible exposure rankings. Overall, results demonstrate that both the missingness mechanism and the choice and specification of imputation strategy substantially influence variable selection in mixture models, underscoring the importance of carefully designed imputation procedures in environmental epidemiology.

## 1. Introduction

### 1.1. Environmental Mixtures

Humans are exposed to various environmental mixtures, including per- and polyfluoroalkyl substances (PFAS), metals, phthalates, phenols, polycyclic aromatic hydrocarbons (PAHs), and more. These arise from water, soil, air, manufacturing, diet, consumer goods, and shared metabolic processes (Carrico et al. 2015; Rosato et al. 2022; Pesenti et al. 2023; Hao et al. 2024). PFAS are commonly found in the environment and tend to bioaccumulate (Rosato et al. 2022), contributing to adverse health outcomes throughout an individual’s life (Carrico et al. 2015; Pesenti et al. 2023). Although widely used in manufacturing and consumer goods (Carrico et al. 2015; Rana 2019; Rosato et al. 2022; Hao et al. 2024) PFAS and other chemicals can detrimentally affect multiple organ systems and are linked to altered birth outcomes, growth, behavior, fertility, cholesterol, and numerous disease processes (Carrico et al. 2015; Rana 2019). Exposure levels vary between individuals and populations, affecting health in children and adults differently (Rana 2019).

Exposure to environmental mixtures and social stressors co-occurs over time. Even when individual components are below the US Environmental Protection Agency (EPA) regulatory limits, mixture components can act synergistically within and between groups (Carrico et al. 2015). Traditional statistical methods typically assess the effects of single pollutants and often fail to capture the dynamics of multiple pollutants, motivating the development of mixture-focused methods over the past decade (Carrico et al. 2015).

### 1.2. Modeling Environmental Mixtures

Classical regression models (e.g. ordinary least squares, logistic regression) struggle with interactive, nonlinear, and non-additive relationships that are common in multipollutant settings (Carrico et al. 2015; Rosato et al. 2022; Pesenti et al. 2023; Hao et al. 2024). Sources and pathways can induce strong correlations between exposures, complicating dose–response assessment and inference (Rana 2019; Keil and Keil 2020). Ignoring synergistic/antagonistic interactions risks bias, misspecification, and misinterpretation, especially under assumptions of linearity and additivity (Carrico et al. 2015; Rana 2019).

Responding to these challenges, the US National Institute of Environmental Health Sciences (NIEHS) emphasized methodological innovation (2012–2017 Strategic Plan) and funded PRIME (Powering Research through Innovative Methods for Mixtures in Epidemiology) (Carrico et al. 2015; Rana 2019; Hao et al. 2024). Since then, many methods have emerged that target different questions (overall/independent/interactive/dose–response effects) with improved precision (Carrico et al. 2015; Rana 2019; Hao et al. 2024). Comparative studies show that no single approach dominates in all scenarios; each has strengths depending on goals such as independent effects, clustered effects, cumulative impact, specification, and multicollinearity (Rana 2019; Keil et al. 2020; Rosato et al. 2022; Pesenti et al. 2023; Hao et al. 2024).

BKMR flexibly models complex, high-dimensional relationships; penalized methods (LASSO, ridge, elastic net) perform well with monotone effects and limited interactions (Pesenti et al. 2023). Random Forests can capture non-linearities and interactions; clustering and principal component analysis (PCA) can group correlated exposures or reduce dimensionality. However, challenges remain: interpretability, simplicity, linearity/additivity assumptions (Pesenti et al. 2023); selection instability under correlated predictors of shrinkage; coefficient shrinkage bias in penalized models (Carrico et al. 2015); and the focus of PCA on predictor correlation rather than outcome association (Carrico et al. 2015). Recent methods include WQS, BKMR, BWQS, Bayesian Additive Regression Trees (BART), G-computation with Super Learner (SLG), Bayesian semiparametric regression (BSR), and BLASSO (Carrico et al. 2015; Pesenti et al. 2023; Hao et al. 2024). WQS builds a weighted index of exposures to test the overall association. Understanding how these methods work in practice is crucial (Hao et al. 2024).

### 1.3. Missing Data

Missing data are a persistent challenge in environmental health research, particularly in the study of chemical mixtures, where multiple correlated exposures are measured simultaneously. In mixture analyses, missing data often stems from concentrations below the limit of detection (LOD), equipment failures, or incomplete monitoring, potentially biasing exposure-response estimates and weakening the identification of high-risk pollutants. Due to the correlated nature of environmental mixtures and their complex joint effects, missing values amplify uncertainty and complicate risk assessments (Carli et al. 2022; Eick et al. 2022).

Current practices for handling missing data in environmental mixtures span a range of statistical and computational approaches. Historically, simple substitution methods, such as replacing values below LOD with LOD divided by the square root of two or zero, were standard; however, these approaches introduce bias and distort correlation structures (Carli et al. 2022; Eick et al. 2022). More advanced single imputation (SI) and multiple imputation (MI) techniques have emerged, including principal component pursuit (PCP), which aids in dimensionality reduction and pattern identification for missing data and values below the LOD (Eick et al. 2022), along with regression-based imputation and reverse Kaplan–Meier methods, though SI often underestimates uncertainty.

MI approaches, particularly MI by Chained Equations (MICE), are now widely applied because they better account for the variability of imputed data. In mixture modeling, Bayesian methods are increasingly being explored. Pseudo-Gibbs and Sequential Full Bayes imputation integrate the imputation step directly into the estimation process, thereby improving power and sensitivity under high missingness (Carli et al. 2022). Outside of epidemiology, environmental monitoring studies also apply Machine Learning (ML) based methods (e.g. neural networks, self-organizing maps, random forests, Long Short-Term Memory [LSTMs]) to impute gaps in near real-time sensor data (Junninen et al. 2004; Zhang and Thorburn 2022). Together, these approaches reflect a shift from ad hoc substitutions toward flexible, model-based imputations that preserve uncertainty and structure critical for reliable inference in environmental mixture studies.

### 1.4. Objectives

This study evaluates and compares the statistical implications of missing-data handling strategies, including mean, median, KNN, MICE, and Amelia, on variable selection performance in environmental mixture models. The analysis focuses on six widely used approaches: WQS, BWQS, Q-gcomp, BKMR, Elastic Net, and LASSO, which represent a range of modeling frameworks for high-dimensional exposure data.

Using extensive Monte Carlo simulations (MCS), we assess how imputation strategies influence the identification of key exposure drivers across different missing data scenarios, including both MAR and MNAR mechanisms. Performance is evaluated using variable selection metrics SE, SP, and FDR under both linear and nonlinear outcome-generating processes.

The study further examines the role of imputation model specification, including the inclusion or exclusion of relevant covariates, in shaping downstream variable selection performance. Because model fit metrics such as AIC are not directly comparable across the diverse modeling frameworks considered, the evaluation is intentionally restricted to variable selection outcomes.

By focusing on variable selection, this work provides a structured framework for understanding how missing data handling affects the identification of important exposures in mixture analyses. The findings offer practical guidance for selecting appropriate imputation strategies and highlight key limitations of commonly used approaches in environmental epidemiology. Among the six mixture techniques studied, WQS regression, BWQS regression, and Q-gcomp are commonly used to investigate the cumulative effect of the mixture components. WQS, originally proposed by Carrico et al. (2015), constructs a weighted index of exposures under a directional assumption, while BWQS extends this framework into a Bayesian setting to allow for uncertainty quantification and greater flexibility (Bobb 2017; Bobb et al. 2018). Q-gcomp further relaxes directional constraints and enables estimation of both overall and component-specific effects, making it suitable for mixtures with heterogeneous directions of association (Renzetti et al. 2023).

In addition to cumulative effect estimation, WQS, BWQS, BKMR, Elastic Net, and LASSO are widely used for variable selection in high-dimensional exposure settings. BKMR is particularly valuable for capturing nonlinear and interactive effects without requiring prior specification of functional forms (Carrico et al. 2015; Bobb 2017; Rosato et al. 2022; Bellavia 2023; Renzetti et al. 2023; Stefano Renzetti 2023; Hao et al. 2024), while Elastic Net and LASSO provide penalized regression frameworks that efficiently identify influential predictors in correlated data structures (Hao et al. 2024). Although extensions of Elastic Net and LASSO can incorporate interaction effects, these variants are beyond the scope of the present study (Hao et al. 2024).

Although several studies have compared mixture modeling approaches using either real-world or simulated data, few have systematically evaluated these models in the context of missing data and imputation strategies. Existing work has emphasized the importance of understanding how pre-processing decisions, such as imputation, influence downstream model performance and inference (Rana 2019; Hao et al. 2024). However, to our knowledge, no study has jointly compared multiple environmental mixture methods while explicitly accounting for the impact of different imputation techniques. This work uses simulation studies to systematically compare environmental mixture models across a range of missing-data handling strategies. This framework enables evaluation of each model’s ability to accurately identify key exposure drivers and maintain robust performance across different missingness mechanisms.

The findings provide important insight into how imputation methods influence model behavior, including variable selection and overall inferential reliability. By highlighting these effects, this study contributes to both environmental health and statistical methodology, offering practical guidance on the appropriate treatment of missing data in environmental mixture analyses.

In addition to the commonly studied MAR settings, real-world environmental data may also exhibit MNAR mechanisms, in which the probability of missingness depends on unobserved values (Rubin 1976; Little and Rubin 2019). While many imputation methods, particularly MI approaches such as MICE, rely on MAR assumptions, violations of these assumptions can substantially impact downstream inference. Furthermore, the specification of the imputation model, including the selection of covariates used in the imputation process, plays a critical role in preserving relationships among variables. Failure to include relevant predictors may introduce bias and reduce the stability of mixture model estimates (Moons et al. 2006; White et al. 2011).

## 2. Materials and Methods

### 2.1. Overview of Environmental Mixture Models

We fit six environmental mixture modeling approaches, including WQS, BWQS, Q-gcomp, BKMR, Elastic Net, and LASSO across complete data, available-case (listwise deletion), and imputed datasets. These methods differ in their ability to accommodate correlated exposures, capture nonlinear relationships, and perform variable selection. Although mixture models can be evaluated using multiple criteria, including overall effect estimation, model fit statistics (e.g. AIC), and predictive performance, direct comparison across methods is not always appropriate due to differences in model structure and underlying assumptions. Therefore, this study focuses on variable importance and selection performance as the primary evaluation metrics. Specifically, we assess each method’s ability to correctly identify influential exposures and distinguish them from non-informative variables under varying missing-data scenarios.

WQS regression constructs a weighted index of quantile exposures:

$$Y_{i}=\beta_{0}+\psi \sum_{j=1}^{p}w_{j}X_{ij}^{q}+Z_{i}^{T}\alpha +\varepsilon_{i},$$

where Y is the outcome, *β*_0_ the intercept, and *ε* the residual error. Each exposure X_j_ is transformed into quantiles $X_{j}^{\left(q\right)}$ to reduce the influence of outliers and harmonize the scales. The weights $w_{j}$ (summing to 1) quantify each exposure’s contribution to the overall mixture index, $Z_{i}^{T}\alpha$ is the linear effect of the vector of covariates, while *ψ* scales the index to the result. WQS is interpretable and effective when exposures act in the same direction; however, it imposes directional homogeneity (i.e. all positive or all negative weights) and struggles with bidirectional or nonlinear effects (Keil and Keil 2020). Current advances allow for the inclusion of both positive and negative directions in the R package, improving flexibility in more complex exposure settings. Despite these advancements, WQS may still be less suited for capturing highly nonlinear relationships compared to methods such as BKMR

BWQS regression extends WQS by relaxing the strict directional assumption and embedding the model in a Bayesian framework:

$$Y_{i}=\beta_{0}+\varphi \sum_{j=1}^{p}w_{j}X_{ij}^{q}+Z_{i}^{T}\alpha +\epsilon_{i}.$$

Here, q_ij_ are quantile-transformed exposures for individual i, w_j_ follows a Dirichlet prior (ensuring they are nonnegative and sum to 1), $Z_{i}^{T}\alpha$ is the linear effect of the vector of covariates, and *ψ* scales the weighted mixture index. Priors allow for the incorporation of uncertainty and prior knowledge. BWQS is more flexible than WQS, allows uncertainty quantification of weights, and can incorporate covariates in a principled way. However, it requires more computational resources and is sensitive to prior specifications. (Elena Colicino 2024).

Quantile g-computation combines the inferential simplicity of WQS with the flexibility of g-computation:

$$Y_{i}=\beta_{0}+\sum_{j=1}^{p}\beta_{j}X_{ij}^{q}++Z_{i}^{T}\alpha +\varepsilon_{i}.$$

Here, $X_{ij}^{\left(q\right)}$ are quantile-transformed exposures, and these are exposure-specific coefficients and $Z_{i}^{T}\alpha$ is the linear effect of the vector of covariates. Q-gcomp allows exposures to have effects in different directions and directly estimates the overall joint effect as the sum of the individual effects *β*_j_. It can also estimate the partial effects of subsets of exposures. Q-gcomp is computationally efficient and provides more nuanced inference, but may be less stable in highly correlated, high-dimensional settings than shrinkage-based methods.

BKMR models the exposure–response relationship through a kernel function:

$$Y_{i}=h\left(X_{i}\right)+Z_{i}^{T}\alpha +\varepsilon_{i}.$$

Here, X_i_ is the vector of exposures for the individual i, h(⋅) is a nonparametric kernel function capturing complex, nonlinear, and interactive effects of the mixture, and $Z_{i}^{T}\alpha$ represents linear effects of covariates. BKMR excels at detecting nonlinearities and interactions without prior specification, offering rich insights into mixture effects. However, it is computationally intensive, sensitive to kernel choice, and less straightforward to interpret than index-based approaches (Bellavia 2023).

Penalized Regression (Elastic Net and LASSO) performs shrinkage and variable selection *via* a penalized least-squares objective:

$$\hat{\beta }=\text{arg}\;\underset{\beta }{\text{min}}\;\{\frac{1}{2n}\sum_{i=1}^{n}{\left(Y_{i}-X_{i}^{⊤}\beta_{z}\right)}^{2}\;+\lambda \left(\frac{1-\eta }{2}\sum_{j=1}^{p}\beta_{j}^{2}+\eta \sum_{j=1}^{p}\left|\beta_{j}\right|\right)\}.$$

Here, *λ* controls overall shrinkage and *η* sets the penalty mix: *η* = 1 yields LASSO (*ℓ*_1_ penalty, allowing some coefficients to be exactly zero), *η* = 0 yields Ridge regression (*ℓ*_2_ penalty, with no exact zeros), and 0 < *η* < 1 corresponds to Elastic Net, which improves stability under strong collinearity by allowing correlated predictors to share information.

In this study, Elastic Net was implemented with *η* = 0.5, representing an equal balance between *ℓ*_1_ and *ℓ*_2_ penalties, which is appropriate for correlated environmental exposures. LASSO was implemented as the special case *η* = 1. The regularization parameter *λ* was selected using 10-fold cross-validation *via* the cv.glmnet function. Specifically, variable selection and coefficient estimation were based on **λ_1_se**, the largest *λ* for which the cross-validated error is within 1 standard error of the minimum. This choice favors a more parsimonious and stable model, which is particularly desirable in high-dimensional and correlated mixture settings.

These methods scale well and effectively identify influential exposures; however, unlike mixture-specific approaches such as WQS, BWQS, or Q-gcomp, they do not estimate a single overall “mixture effect.” In addition, variable selection can be unstable or overly aggressive under extreme collinearity, particularly for pure LASSO.

In summary, WQS, BWQS, and Q-gcomp are mixture-specific approaches that emphasize interpretability of cumulative effects; BKMR is well-suited for detecting nonlinear and interaction effects; and LASSO and Elastic Net are general-purpose penalized regression methods that excel in variable selection and high-dimensional settings, although they do not directly estimate an overall mixture effect. Together, these models provide complementary insights into environmental mixture analyses, balancing interpretability, flexibility, stability, and computational efficiency across both continuous and binary outcomes under consistent mixture and missingness scenarios.

### 2.2. Overview of Missing Data Mechanisms

Missingness is prevalent in real-world datasets, such as surveys and Electronic Health Records (EHRs). It can degrade the performance of ML/statistical models that assume complete data (King et al. 2001; Chowdhury et al. 2017; Kim et al. 2019; Shadbahr et al. 2023; Mahmood et al. 2024). Causes include survey dropout, partial responses, mishandling, recording errors, and sampling bias, and contribute to prediction errors (Faraway 2014; Chowdhury et al. 2017; Shadbahr et al. 2023). Researchers often impute missing values or apply listwise deletion prior to fitting models (Faraway 2014; Kim et al. 2019; Shadbahr et al. 2023), and model performance typically worsens as missingness increases (Shadbahr et al. 2023). The key questions concern how missingness rates and imputation approaches affect model performance and the recovery of the underlying data-generating signal using metrics such as Root Mean Squared Error (RMSE), Mean Absolute Error (MAE), R-squared, and deviation (Shadbahr et al. 2023).

Let D = {Y, X} denote the complete data matrix, where Y represents the outcome and X the set of predictors. Let M be a missingness indicator matrix defined as:

$$M_{ij}=\left\{\begin{array}{c} 1,\;\mathrm{if}\;D_{ij}\;\mathrm{is observed}\\ 0,\mathrm{if}\;D_{ij}\;\mathrm{is missing} \end{array}.\right.$$

We partition the data as D = {D_obs_, D_mis_} corresponding to the observed and missing components (King et al. 2001).

Following Rubin’s framework (Rubin 1976) and subsequent developments (Rubin 1976; King et al. 2001; Van Buuren and Groothuis-Oudshoorn 2011; Li et al. 2024). Missing-data mechanisms are defined in terms of the conditional distribution of the missingness indicator M.

#### 2.2.1. Missing Completely at Random (MCAR)


$$Pr\left(M|D_{obs},D_{mis}\right)=Pr(M).$$

Missingness is independent of both observed and unobserved data. Under MCAR, complete-case analysis yields unbiased estimates but may reduce efficiency (Van Buuren and Groothuis-Oudshoorn 2011; Faraway 2014).

#### 2.2.2. Missing at Random (MAR)


$$Pr\left(M|D_{\text{obs}},D_{\text{mis}}\right)=Pr\left(M|D_{\text{obs}}\right).$$

Missingness depends only on observed data, and valid inference can be obtained using appropriate imputation or likelihood-based methods.

#### 2.2.3. Missing Not at Random (MNAR)


$$Pr\left(M|D_{\text{obs}},D_{\text{mis}}\right)\;\text{depends on}\;D_{\text{mis}}.$$

Missingness depends on unobserved values and requires explicit modeling of the missing data mechanism.

These definitions provide the theoretical foundation for evaluating how different imputation strategies perform under varying missing-data mechanisms.

### 2.3. Missing Data Handling Techniques

We evaluated seven datasets for each simulation: (i) Complete data (ii) Listwise deletion, (iii) Mean imputation, (iv) Median imputation, (v) KNN, (vi) MI *via* MICE with *m* = 5, and (vii) MI *via* Amelia (EMB), with *m* = 5.

#### 2.3.1. Listwise Deletion

Complete case analysis removes any record with missing values (King et al. 2001; Van Buuren and Groothuis-Oudshoorn 2011). It is simple and widely available, but it discards observations with missing values and can lead to inconsistent sample sizes across analyses. Under MCAR, complete-case analysis yields unbiased estimates but is typically inefficient due to loss of information. Under MAR, estimates may be biased, particularly for means, correlations, and descriptive statistics, unless the missingness mechanism depends only on the covariates included in the model; in that case, regression coefficients remain unbiased. In general, however, listwise deletion is not recommended due to potential bias and reduced statistical power (Van Buuren and Groothuis-Oudshoorn 2011).

#### 2.3.2. Imputation

Imputation replaces missing values using the observed data structure. Many methods assume MAR (Van Buuren and Groothuis-Oudshoorn 2011; Li et al. 2024). Available approaches include simple imputation (mean and median), regression imputation, expectation–maximization (EM), MI, KNN, clustering, tree-based methods such as Classification and Regression Trees (CART) and Random Forests (e.g. MissForest), and deep generative approaches such as Generative Adversarial Imputation Networks (GAIN) and Missing Importance-Weighted Autoencoders (MIWAE) (Shadbahr et al. 2023; Li et al. 2024). In this study, we focus on three classes of imputation methods: (i) simple deterministic imputation (mean, median), (ii) a distance-based ML approach (KNN), and (iii) MI methods (MICE and Amelia). Simple methods are easy to apply but may be biased and understate uncertainty (Faraway 2014).

##### 2.3.2.1. Simple imputation techniques.

Simple (single-value) imputation replaces each missing observation with a single estimated value, enabling standard statistical analyses to be performed on the completed dataset (Fielding et al. 2008). SI encompasses a range of approaches, including summary-statistic replacement methods (e.g. mean, median, or mode imputation) and model-based methods such as regression or k-nearest neighbors.

In this study, we include both summary-statistic methods (mean and median) and a distance-based method, KNN. These approaches remain widely used in applied analyses because of their computational simplicity and ease of implementation, although they have well-known limitations. ***Mean:*** Replace missing values with the variable’s mean value. It is fast but shrinks variance and biases correlations, distorting the structure of the mixture (Junninen et al. 2004; Zhang and Thorburn 2022). **Median** imputation replaces missing values with the median value. It is more robust to skew/outliers, but still deterministic, and may mask cross-variable associations (Junninen et al. 2004; Carli et al. 2022). **Mode** is often used with categorical variables. It assigns the most frequent category, preserving marginal distribution, but this approach can underestimate variability and weaken associations (Eick et al. 2022; Zhang and Thorburn 2022). In general, the mean and median are easy to calculate but can attenuate effects and understate standard errors. MI or ML-based methods are generally preferred for correlated environmental mixtures (Eick et al. 2022; Zhang and Thorburn 2022).

***KNN*** imputation, an ML-based approach, replaces missing values using information from similar observations identified by distance metrics (Troyanskaya et al. 2001; Beretta and Santaniello 2016). Unlike summary-statistic methods, KNN leverages multivariate relationships in the data, thereby improving recovery of the underlying structure in correlated environmental mixtures (Junninen et al. 2004). KNN was implemented using a distance-based approach with *k* = 5 nearest neighbors, using all available variables. However, KNN remains an SI method and does not account for uncertainty in the imputation process, potentially leading to biased inference and underestimated variability (Van Buuren 2018). Despite its increasing use in environmental and biomedical data contexts, its performance relative to traditional statistical imputation methods in mixture modeling settings remains underexplored.

Because these approaches do not account for uncertainty in the imputation process, they may underestimate variability and attenuate associations among variables. Consequently, more advanced methods, such as MI or ML-based approaches, are often preferred for complex, correlated data structures. Despite these limitations, simple, distance-based imputation methods, such as KNN, are often included in simulation studies as baseline comparators.

##### 2.3.2.2. Multiple imputation (MI).

MI is recommended for complex missing patterns across multiple variables (King et al. 2001; Van Buuren and Groothuis-Oudshoorn 2011; Van Buuren 2018). Two major frameworks are Joint Modeling (JM) and Fully Conditional Specification (FCS). The study included MICE and Amelia imputation in multiple data scenarios, under the MVNORM and MVT distributions for continuous and binary outcome variables with linear or non-linear relationships.

***MICE*** was implemented using FCS, where each variable with missing values is imputed using a regression model conditional on all other variables. Suppose Y is partially observed from a p-variate distribution P(Y∣θ). FCS samples from a sequence of univariate conditional models (Van Buuren and Groothuis-Oudshoorn 2011):

$$P\left(Y_{1}|Y_{-1},\theta_{1}\right),\ldots ,P\left(Y_{p}|Y_{-p},\theta_{p}\right),$$

Iterating a Gibbs-like scheme. In iteration t,

$$\theta_{1}^{\text{*}(\text{t})}∼\text{P}\left(\theta_{1}|{\text{Y}}_{1}^{\text{obs}},{\text{Y}}_{2}^{\left(\text{t}-1\right)},\ldots ,{\text{Y}}_{\text{p}}^{\left(\text{t}-1\right)}\right),{\text{Y}}_{1}^{\text{*}(\text{t})}\;∼\text{P}\left({\text{Y}}_{1}|{\text{Y}}_{1}^{\text{obs}},{\text{Y}}_{2}^{\left(\text{t}-1\right)},\ldots ,{\text{Y}}_{\text{p}}^{\left(\text{t}-1\right)},\theta_{1}^{\text{*}(\text{t})}\right),:\theta_{\text{p}}^{\text{*}(\text{t})}\;∼\text{P}\left(\theta_{\text{p}}|{\text{Y}}_{\text{p}}^{\text{obs}},{\text{Y}}_{1}^{\left(\text{t}\right)},\ldots ,{\text{Y}}_{\text{p}-1}^{\left(\text{t}\right)}\right),{\text{Y}}_{\text{p}}^{\text{*}(\text{t})}\;∼\text{P}\left({\text{Y}}_{\text{p}}|{\text{Y}}_{\text{p}}^{\text{obs}},{\text{Y}}_{1}^{\left(\text{t}\right)},\ldots ,{\text{Y}}_{\text{p}}^{\left(\text{t}\right)},\theta_{\text{p}}^{\text{*}(\text{t})}\right).$$

Convergence is typically reached in 10–20 iterations (Van Buuren and Groothuis-Oudshoorn 2011).

For a specified distribution, relationship, or outcome, MICE imputation is performed on the data with exposure-only, outcome-only, or both exposure and outcome missingness, using *m* = 5 imputation, before fitting the models with the MICE-imputed datasets. For each simulation iteration, we combined the pooled average of 5 estimates using Rubin’s rule, performed variable selection, and evaluated characteristics using model-specific estimates. We then pooled these estimates to obtain SE, SP, and FDR, and determined significance using model-specific metrics. The pooling before classification ensures that the uncertainty introduced by imputing missing data is propagated into selection decisions and performance metrics.

We specified variable-specific imputation models within the FCS framework as follows: continuous variables (exposures and covariates such as age and income, as well as continuous outcomes) were imputed using predictive mean matching (PMM), while binary variables (e.g. gender and binary outcomes) were imputed using logistic regression. All variables included in the analysis models were incorporated as predictors in the imputation models unless otherwise specified.

***Amelia*** was implemented using the expectation–maximization with bootstrap (EMB) algorithm, which assumes approximate multivariate normality of the data (Honaker et al. 2011). The EMB approach generates bootstrap samples, applies the EM algorithm to estimate model parameters and impute missing values, and incorporates uncertainty through repeated resampling to produce multiple imputed datasets. In this study, Amelia was applied with default settings, including all exposures, covariates, and outcome variables as predictors in the imputation model, and *m* = 5 imputations were generated for each dataset.

##### 2.3.2.3. Pooling of estimates.

Parameter estimates were pooled across imputations using Rubin’s rules (King et al. 2001; Guidi et al. 2021). For m = 5 imputations, let ${\overset{ˆ}{q}}_{j}$ denote the estimate and $SE\left({\overset{ˆ}{q}}_{j}\right)$ it’s standard error from the j-th imputed dataset (*j* = 1, …, m). The pooled estimate is given by:

$$\bar{q}=\frac{1}{m}\sum_{j=1}^{m}{\overset{ˆ}{q}}_{j},$$

with the within-imputation variance

$$\bar{U}=\frac{1}{m}\sum_{j=1}^{m}SE{\left({\overset{ˆ}{q}}_{j}\right)}^{2},$$

and between-imputation variance

$$B=\frac{1}{m-1}\sum_{j=1}^{m}{\left({\overset{ˆ}{q}}_{j}-\overset{‾}{q}\right)}^{2}.$$

The total variance is then:

$$T=\bar{U}+\left(1+\frac{1}{m}\right)B.$$

To summarize results across Monte Carlo simulations, pooled estimates were first obtained within each simulation using Rubin’s rules. These simulation-specific estimates were then aggregated across simulations to evaluate overall performance (King et al. 2001; Faraway 2014). For parameter *β*_j_, let ${\overset{ˆ}{\beta }}_{ij}$ denote the pooled estimate from simulation i (*i* = 1, …, N). The overall estimate is computed as:

$${\overset{ˆ}{\beta }}_{j}=\frac{1}{N}\sum_{i=1}^{N}{\overset{ˆ}{\beta }}_{ij},$$

with variability summarized using the empirical variance of ${\overset{ˆ}{\beta }}_{ij}$ across simulations. Binary variables were specified as nominal to account for their categorical nature.

### 2.4. Performance Metrics

We evaluated variable selection performance using SE, SP, and FDR. Let S denote the number of simulation replicates. For each replicate *s* = 1, …, S, exposures were classified as: true positives (TP; causal exposures correctly selected), false negatives (FN; causal exposures not selected), false positives (FP; null exposures incorrectly selected), and true negatives (TN; null exposures correctly excluded). Performance metrics were defined as:

$$\text{Sensitivity}=\frac{1}{S}\sum_{s=1}^{S}\frac{{\mathrm{TP}}_{s}}{{\mathrm{TP}}_{s}+{\mathrm{FN}}_{s}},$$

$$\text{Specificity}=\frac{1}{S}\sum_{s=1}^{S}\frac{{\mathrm{TN}}_{s}}{{\mathrm{TN}}_{s}+{\mathrm{FP}}_{s}},$$

$$\mathrm{FDR}=\frac{1}{S}\sum_{s=1}^{S}\frac{{\mathrm{FP}}_{s}}{{\mathrm{TP}}_{s}+{\mathrm{FP}}_{s}}.$$

These metrics quantify each method’s ability to correctly identify true exposures (SE), avoid selecting null exposures (SP), and control the FDR among selected exposures (Hao et al. 2024).

Across all simulation settings, X_1_–X_5_ were designated as true exposures and X_6_–X_10_ as null exposures. The underlying effect structure (linear or nonlinear) was determined by the outcome-generating mechanism.

### 2.5. Variable Selection Criteria

Because each mixture modeling method operates under a distinct statistical framework, variable selection criteria were defined in a method-specific manner:

#### WQS:

Selection based on bootstrap-derived weights and 95% confidence intervals. An exposure was considered selected if its confidence interval did not include zero.

#### BWQS:

Selection based on posterior mean weights and credible intervals. A threshold of ≥ 0.05 on the posterior mean was used to avoid assigning importance to negligible weights.

#### Q-gcomp:

Exposures were modeled as quantile predictors and considered selected if the regression coefficient satisfied *p* < 0.05.

#### BKMR:

Variable importance was assessed using posterior inclusion probabilities (PIPs). Following convention, thresholds of PIP ≥ 0.5 for continuous outcomes and ≥ 0.1 for binary outcomes were used to declare selection.

#### Elastic Net and LASSO:

Penalized regression coefficients were obtained using cross-validation to select the tuning parameter *λ*_1se_. Exposures were considered selected if $|\overset{ˆ}{\beta }|\geq 0.05$, reflecting non-negligible contributions after penalization.

#### Interpretation:

**Figure 1.**
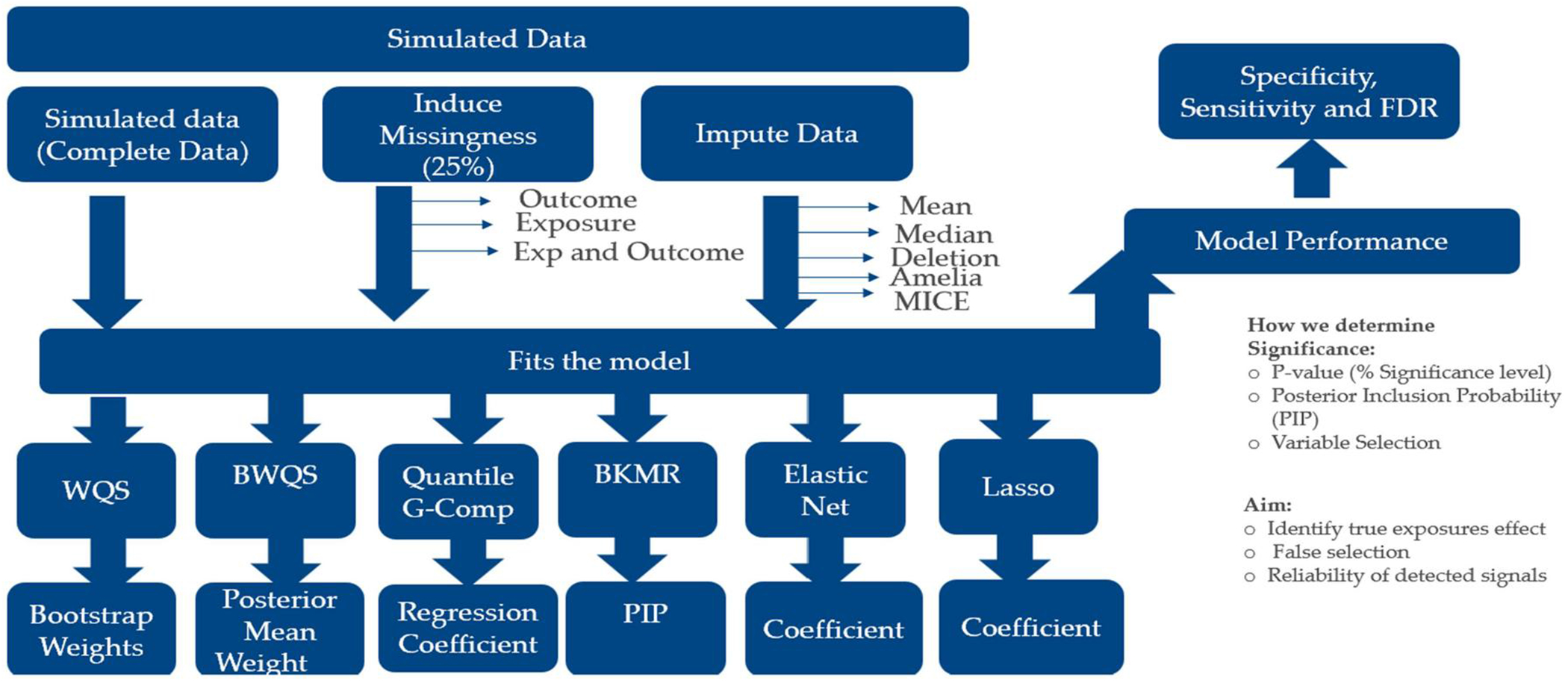
Process overview of the research methods: data generation, inducing missingness, applying missing data techniques (including pooling), and evaluating model performance.

These selection rules provide a harmonized yet method-appropriate framework for evaluating variable selection performance across models. While thresholds differ by method, they reflect standard practice within each modeling framework and ensure comparability of SE, SP, and FDR across simulation settings. The framework is illustrated in Figure 1.

## 3. Simulation Study

We conducted a simulation study to evaluate the performance of environmental mixture models under varying exposure distributions, exposure–response relationships, and missing-data mechanisms.

### 3.1. Data Generation

For each Monte Carlo iteration, we generated *n* = 1000, observations with *p* = 10*p* = 10 exposures (X_1_, …, X_10_), where X_1_ – X_5_ are active (non-zero effects) and X^6^ – X_10_ are null. We conducted 100 Monte Carlo replicates. In addition, three covariates, age, gender, and income, were generated for use in adjustment and in the missingness mechanisms.

#### 3.1.1. Exposure Variables

We simulated exposures under two distributions: (i) a multivariate normal (MVNORM) distribution and (ii) a multivariate t (MVT) distribution with *ν* = 10 degrees of freedom. Both distributions share a common correlation structure, while the MVT introduces heavier tails to reflect extreme values commonly observed in environmental data.

Let 1 be the p-dimensional vector of ones and I_p_ the p × p identity matrix. The covariance structure is defined using an equicorrelated matrix:

$$\Sigma_{\rho }=(1-\rho )I_{p}\;+\;\rho 11^{⊤},\;p=10,$$

so that $Var\left(X_{j}\right)=1$ and $Cor\left(X_{j},X_{l}\right)=\rho$ for j≠1. This matrix is positive definite for −1/(p−1p−1)<ρ<1: We set *ρ* = 0.2, representing moderate inter-exposure correlation.

For each subject *i* = 1, …, n, exposures were generated as:

$$X_{i}∼\left\{\begin{array}{cc} N_{p}\left(0,\Sigma_{\rho }\right), & \left(MVNORM\right)\\ t_{\nu =10,p}\left(\delta =0,\Sigma_{\rho }\right), & \left(MVT\right) \end{array}\right.,$$

Under the MVT distribution, the covariance is inflated by a factor of $\frac{v}{v-2}=1.25$, resulting in heavier-tailed exposures with greater marginal variability than in the MVNORM case. This formulation allows us to evaluate model robustness under departures from normality.

#### 3.1.2. Covariates

The covariates were generated independently as:

$$\text{Age}∼\text{N}\left(50,10^{2}\right),\;\text{gender}∼\text{Bernoulli}(0.5),\;\text{income}∼\text{N}\left(50,000,10,000^{2}\right).$$

All exposure variables (X_1_ – X_10_) and covariates were included in most models. In selected scenarios, age and gender were included while income was excluded to evaluate the impact of imputation model specification. Specifically, to assess robustness to imputation model misspecification, income was excluded from selected imputation models while retained in the data-generating process.

#### 3.1.3. Outcome Variable

We consider both Gaussian (continuous) and logistic (binary) outcomes.

##### 3.1.3.1. Continuous outcome.

The continuous outcome was generated as:

$$Y_{i}=\mu_{i}+\varepsilon_{i},\;\varepsilon_{i}∼N\left(0,\sigma_{e}^{2}\right),\sigma_{e}=sd.$$

with two exposure–response structures:

Linear:

$$Linear:\;\mu_{i}=0.5\;X_{i1}+0.3\;X_{i2}-0.2\;X_{i3}+0.1\;X_{i4}-0.05\;X_{i5}.$$

Nonlinear:

$$\mu_{i}=0.5\;e^{X_{i1}}\;+\;0.3\sqrt{\left|X_{i2}\right|}-0.2log\left(1+\left|X_{i3}\right|\right)+0.1\;X_{i4}^{2}-0.05\;X_{i5}^{3}.$$

$$\text{Continuous}\;:\;{\text{Y}}_{\text{i}}\;=\;\mu_{\text{i}}+\varepsilon_{\text{i}},\;\varepsilon_{\text{i}}∼\text{N}(0,1).\text{Binary}\;:\;{\text{Y}}_{\text{i}}\;∼\text{Bernoulli}\left({\text{p}}_{\text{i}}\right),\;{\text{p}}_{\text{i}}={\text{logit}}^{-1}\left(\mu_{\text{i}}\right).$$

Given X_i_, we define a linear predictor *μ*_i_ and we add homoskedastic Gaussian noise:

##### 3.1.3.2. Binary outcome generator.

A binary result is generated through a logistic link with a linear or complex nonlinear structure (Hao et al. 2024). Let *μ*_i_ denote the linear predictor for the subject i

$$Y_{i}∼Bernoulli\left(p_{i}\right),p_{i}\;=\;{logit}^{-1}\left(\mu_{i}\right)=\frac{1}{1+exp\left(-\mu_{i}\right)},$$

Again, we consider linear and nonlinear exposure–response structures; the linear form is

$$\mu_{i}=0.5\;X_{i1}+0.3\;X_{i2}-0.2\;X_{i3}+0.1\;X_{i4}-0.05\;X_{i5},$$

and the non-linear specification used is:

$$\mu_{\text{i}}={\text{X}}_{\text{i}1}\;I\left\{{\text{X}}_{\text{i}1}>0\right\}+{\text{e}}^{{\text{X}}_{\text{i}2}}\;+\;\left|{\text{X}}_{\text{i}3}\right|+{\text{X}}_{\text{i}4}^{2}+{\left({\text{X}}_{\text{i}5}+1\right)}^{2}\;+{\text{X}}_{\text{i}1}{\text{e}}^{{\text{X}}_{\text{i}2}}+{\text{X}}_{\text{i}3}\;I\left\{{\text{X}}_{\text{i}1}>0\right\}+{\text{X}}_{\text{i}2}\frac{{\text{X}}_{\text{i}1}^{2}}{{\left({\text{X}}_{\text{i}1}+1\right)}^{2}}+{\text{e}}^{{\text{X}}_{\text{i}2}}{\text{X}}_{\text{i}3}\;+{\text{X}}_{\text{i}3}\left({\text{X}}_{\text{i}5}^{2}+{\text{X}}_{\text{i}3}^{2}+{\left({\text{X}}_{\text{i}5}+1\right)}^{2}\right).$$

We also add element-wise jitter N(0, 10^−12^) to X_i_.

All simulation components were implemented directly in R, and the data-generating mechanisms described above correspond exactly to the functions used in the simulation code.

### 3.2. Missing Data Mechanism

We induced missingness under MAR and MNAR mechanisms. Let Z_i_ = (age_i_, gender_i_, income_i_) denote the vector of fully observed covariates for subject i.

#### 3.2.1. MAR Mechanism

Under the MAR mechanism, missingness depended only on observed covariates. We defined a subject-specific missingness propensity as

$$\eta_{i}=0.1\;{\text{age}}_{i}+0.2\;{\text{gender}}_{i}-10^{-5}\;{\text{income}}_{i},$$

with corresponding logistic transformation

$$g\left(\eta_{i}\right)={\left\{1+exp\left(-\eta_{i}\right)\right\}}^{-1}.$$

For a chosen scaling factor r ∈ (0, 1), the missingness probability for the subject i was defined as

$$p_{i}=r\times g\left(\eta_{i}\right).$$

Thus, under MAR, missingness probabilities vary across subjects but are shared within a subject across exposures. Older age and male sex increase p_i_, whereas higher income decreases it.

#### 3.2.2. MNAR Mechanism

Under the MNAR mechanism, missingness depended on both observed covariates and the exposure values themselves. For each exposure X_ij_, we first standardized the exposure values:

$$X_{ij}^{*}=\frac{X_{ij}-{\overset{‾}{X}}_{j}}{sd\left(X_{j}\right)}.$$

We then defined an exposure-specific missingness propensity:

$$\eta_{ij}^{MNAR}=0.8\;X_{ij}^{*}+0.1\;{\text{age}}_{i}+0.2\;{\text{gender}}_{i}-10^{-5}\;{\text{income}}_{i},$$

with corresponding probability

$$p_{ij}=r\times {\left\{1+exp\left(-\eta_{ij}^{MNAR}\right)\right\}}^{-1}.$$

Unlike MAR, missingness now varies across both subjects and exposures. Higher exposure values increase the probability of missingness, inducing dependence on unobserved data.

#### 3.2.3. Missingness Scenarios

We considered three missingness scenarios:

##### 3.2.3.1. Exposure-only missingness.

For each subject i and exposure j, draw U_ij_ ~ Unif(0, 1). Set

$$X_{ij}=\text{NA if}\;U_{ij}<p_{ij},$$

where p_ij_ = p_i_ under MAR and p_ij_ is exposure-specific under MNAR. This induces nonmonotone missingness across exposures.

##### 3.2.3.2. Outcome-only missingness.

For each subject i under draw U_i_ ~ Unif(0, 1). Set

$$Y_{i}=\text{NA if}\;U_{i}<p_{i},$$

while exposures remain fully observed.

##### 3.2.3.3. Exposure and outcome missingness.

Combine the two mechanisms above using independent uniform draws. Conditional on Z_i_, Exposure and outcome missingness are independent, but marginal dependence arises through shared covariate effects.

#### 3.2.4. Missingness Rate Calibration

The logistic transformation ensures g(η)∈(0,1), so p∈(0,r). The expected missingness rate satisfies

$$E\left[p_{i}\right]=r\;E\left[g\left(\eta_{i}\right)\right],$$

which is typically less than r.

To target a desired missingness rate $\bar{r}$, we calibrate an intercept a such that

$$\frac{1}{n}\sum_{i=1}^{n}g\left(\alpha +\eta_{i}\right)=\bar{r},$$

and redefine

$$p_{i}=g\left(\alpha +\eta_{i}\right).$$

### 3.3. Reproducibility

All analyses were implemented directly in R (version 4.5.1) using functions that correspond exactly to the formulations described above. A fixed random seed was used to ensure reproducibility across simulations. Unless otherwise specified, the nominal missingness rate was set to 25%, with additional scenarios at 10% and 5% for SE analyses. Detailed simulation and analysis code, including parameter settings, model-specific extraction rules, and post-processing workflows, has been provided in the GitHub repository: https://github.com/ysboafo-coder/Evaluating-Missing-Data-Imputation-Strategies-for-Environmental-Mixture-Models-An-Empirical-Study. The GitHub framework includes a unified pipeline covering all missing-data scenarios, outcome types, exposure distributions, and mixture methods evaluated in this study.

## 4. Results

This section summarizes results for both continuous and binary outcomes. Six mixture modeling methods (WQS, BWQS, Q-gcomp, BKMR, Elastic Net, LASSO) were evaluated across three missingness scenarios (exposure, outcome, and joint missingness), under MVNORM and MVT exposure distributions, and under both linear and nonlinear relationships. Model performance was assessed using SE, SP, and FDR.

### 4.1. Continuous Outcomes

Under MVNORM, BKMR, and Q-gcomp consistently demonstrated the strongest performance. BKMR maintained high SE (≈0.90–0.94) and SP (≈0.82–0.93) under multiple imputation, while Q-gcomp achieved the highest SE (≈0.97). Elastic Net and LASSO remained highly specific but less sensitive, while WQS and BWQS showed moderate performance that varied. MICE and Amelia provided the most stable results, while SI approaches (mean, median, KNN) were more variable.

**Figure 2.**
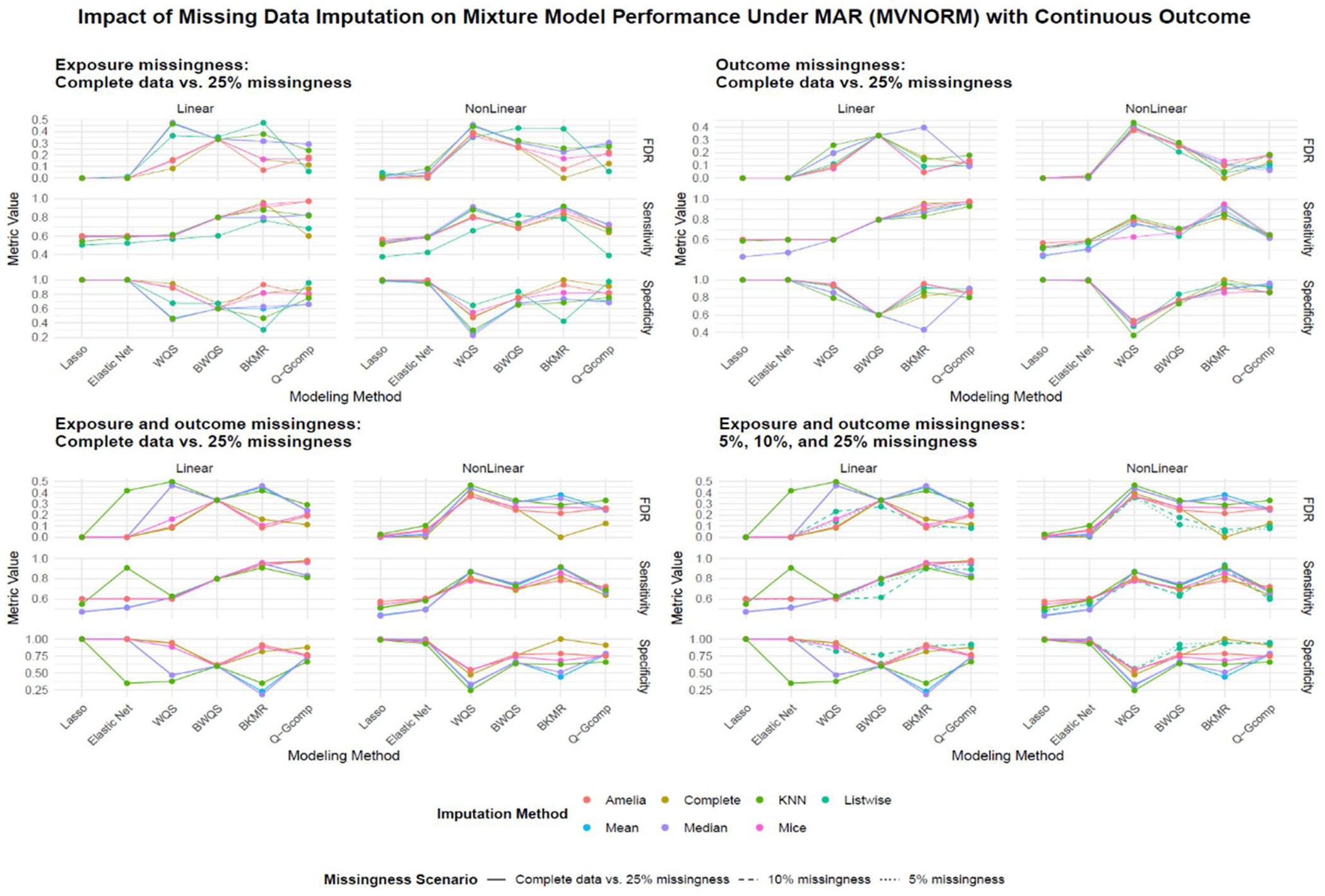
Performance of mixture models under MAR with multivariate normal exposures and a continuous outcome. Top panels: exposure-only and outcome-only missingness. Bottom panels: combined exposure and outcome missingness comparing complete data with 25% missingness and with multiple missingness rates (5%, 10%, and 25%).

**Figure 3.**
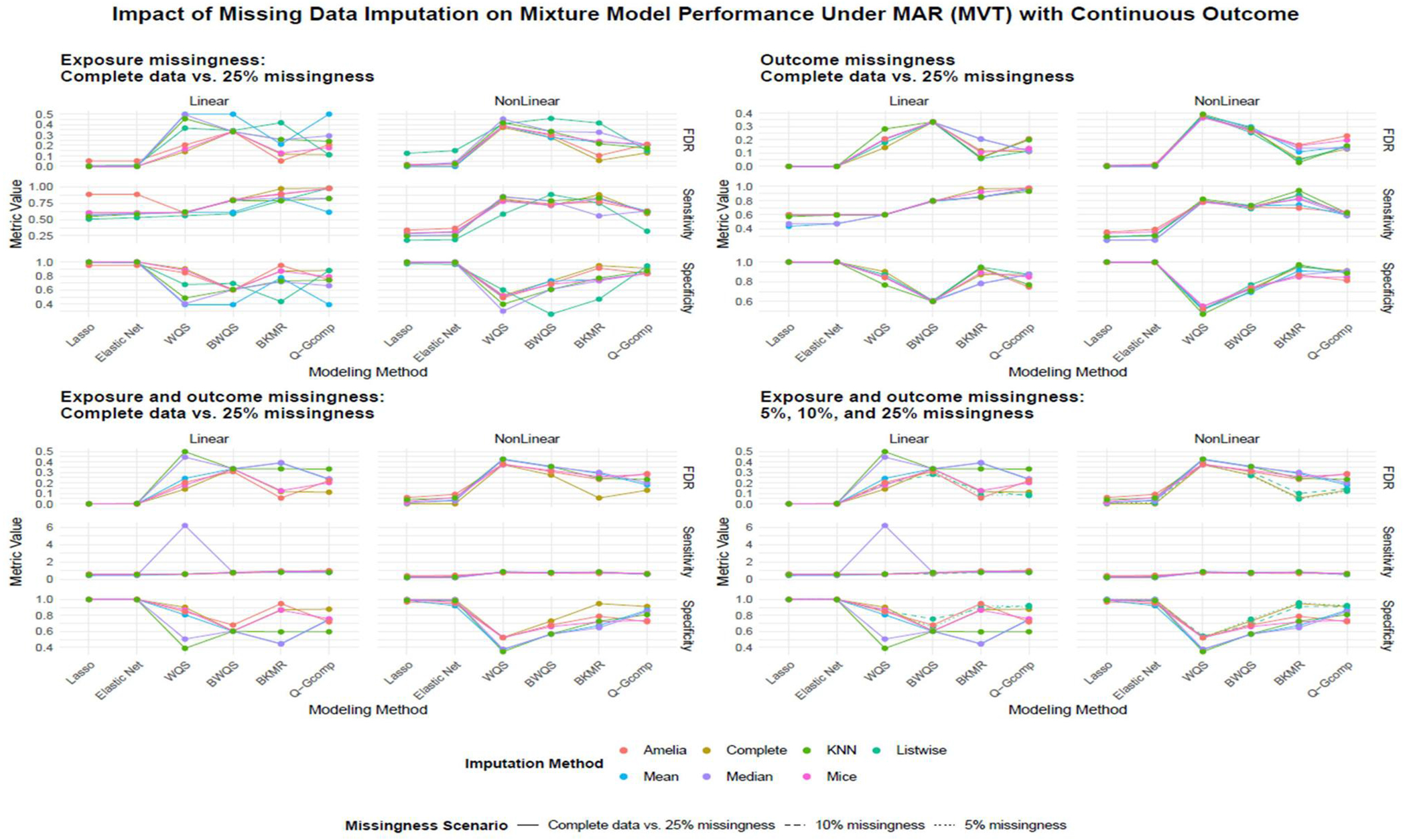
Performance of mixture models under MAR with MVT exposures and a continuous outcome under linear and nonlinear relationships. Panel structure mirrors Figure 2.

Under nonlinear settings, BKMR remained the most robust method, with Q-gcomp as a competitive alternative. Similar patterns were observed across missingness mechanisms. Results under MVT exposures were consistent with MVNORM, with BKMR and Q-gcomp remaining the most reliable methods. Figures 2 and 3 illustrate the results for MVNORM and MVT under the MAR assumption for a continuous outcome.

Tables 1–4 summarize detailed results by distribution, missingness mechanism, and relationship type.

### 4.2. Binary Outcomes

Under MVNORM, Q-gcomp provided the most consistent balance between SE, SP, and FDR. Elastic Net and LASSO minimized false discoveries but at the expense of SE. WQS and BKMR achieved high SE but showed poor SP and elevated FDR. MI yielded the most stable performance, while SI approaches (including KNN) were more variable.

**Figure 4.**
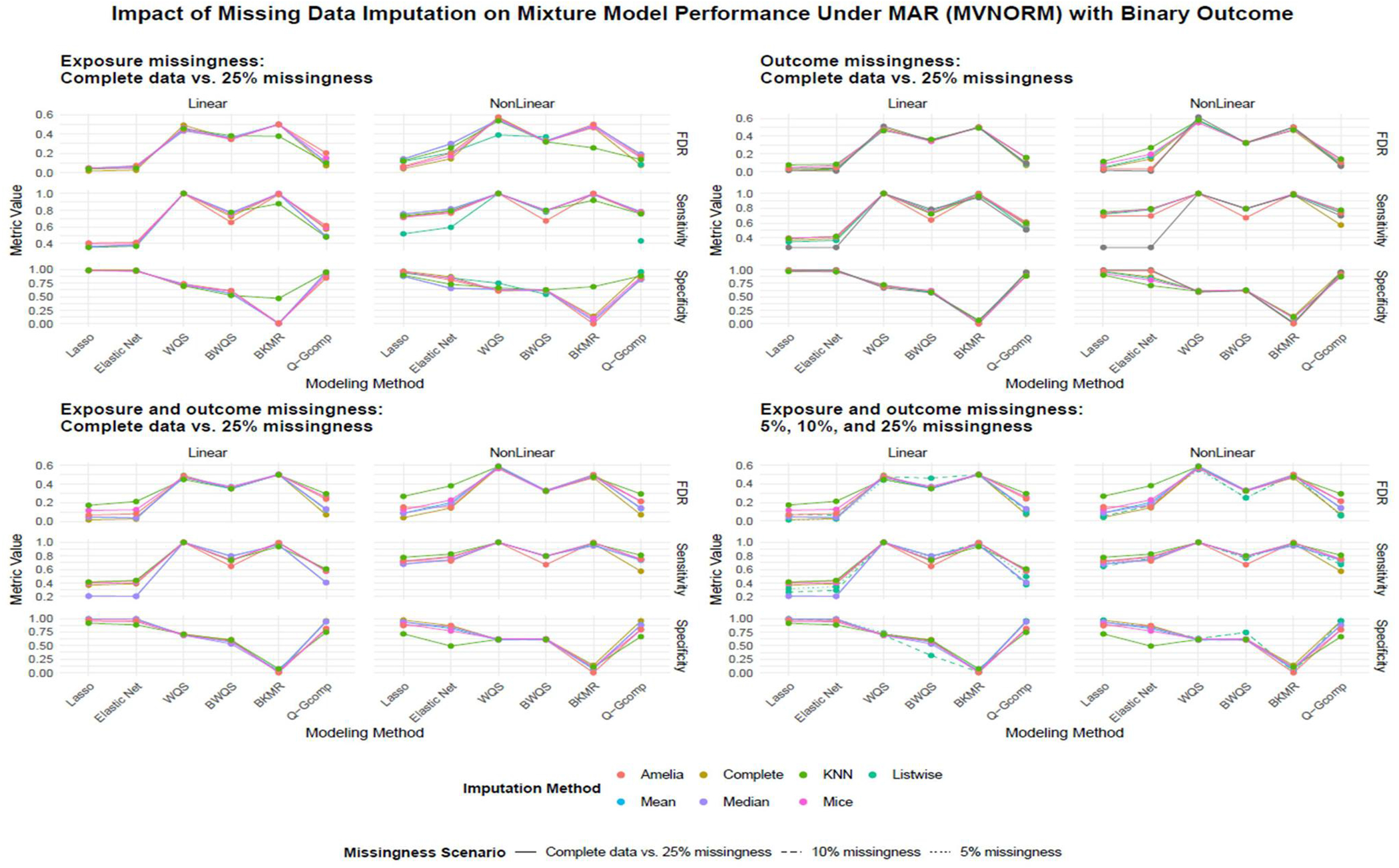
Performance of mixture modeling methods under MAR with multivariate normal exposures (binary outcome). Top row: exposure-only and outcome-only missingness. Bottom row: exposure and outcome comparisons (Complete vs 25%; all rates 5–25%). Each panel summarizes Sensitivity, Specificity, and FDR by method, split by linear vs. nonlinear relationships and imputation/treatment type.

**Figure 5.**
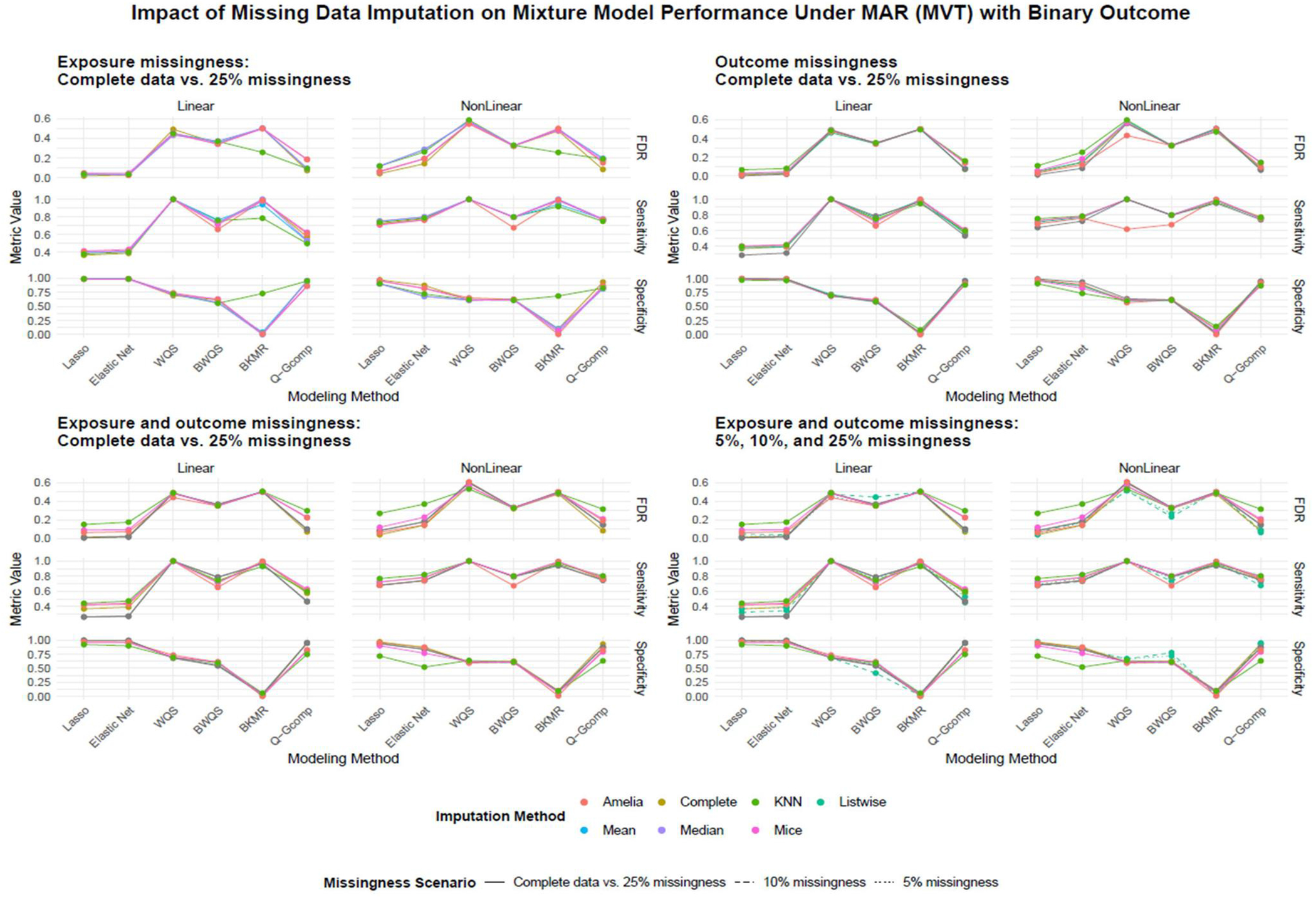
This figure summarizes model performance under MAR with multivariate T (MVT) exposures and binary outcomes. Top panels: Exposure-only and outcome-only missingness. Bottom panels: Combined exposure with outcome missingness (Complete vs 25%; MR = 5–25%).

Under MVT exposures, findings were consistent. Q-gcomp maintained the best balance across metrics, while Elastic Net/LASSO remained conservative and WQS/BKMR exhibited high SE with poor SP. Figures 4 and 5 illustrate the results for MVNORM and MVT under the MAR assumption for a binary outcome.

Tables 5–8 summarize the handling of missing data and model performance across distributions and outcome relationships. Listwise deletion was not evaluated at the 25% missingness rate for datasets with both exposure and outcome missingness because applying a 25% independent missingness probability across all variables (10 exposures and one outcome) reduces the expected complete-case sample size to near zero. Specifically, the probability of a fully observed case is 0.75^11^ ≈ 4.2%, resulting in an insufficient sample size for stable model estimation. To ensure computational feasibility and maintain meaningful comparisons, the maximum missingness rate for listwise deletion scenarios was therefore restricted to 10% and 5% in datasets with concurrent exposure and outcome missingness.

### 4.3. Summary of Findings

Across both outcome types and exposure distributions, model performance was primarily driven by the interaction between modeling method and imputation strategy. Q-gcomp consistently balanced SE, SP, and FDR, while BKMR performed best for continuous outcomes but less reliably for binary outcomes. MI methods provided the most stable results, while SI approaches were more variable. This study demonstrates that mixture model performance is strongly influenced by the interaction between modeling approach, imputation method, and missingness mechanism. Q-gcomp consistently provided the most balanced performance and remained robust under MNAR and imputation model misspecification. BKMR performed strongly under MAR but deteriorated under MNAR and for binary outcomes. Elastic Net and LASSO prioritized SP and false discovery control, while WQS and BWQS showed variable performance.

Violations of the MAR assumption substantially degraded performance, particularly in SP and FDR. MI methods were effective under MAR but less stable under MNAR. Additionally, excluding relevant covariates from imputation models introduced instability, emphasizing the importance of proper imputation design.

Overall, the missingness mechanism and the specification of the imputation model play critical roles in the performance of mixture modeling, with important implications for environmental epidemiology research.

### 4.4. Effect of Missingness Mechanism (MAR vs. MNAR)

**Figure 6.**
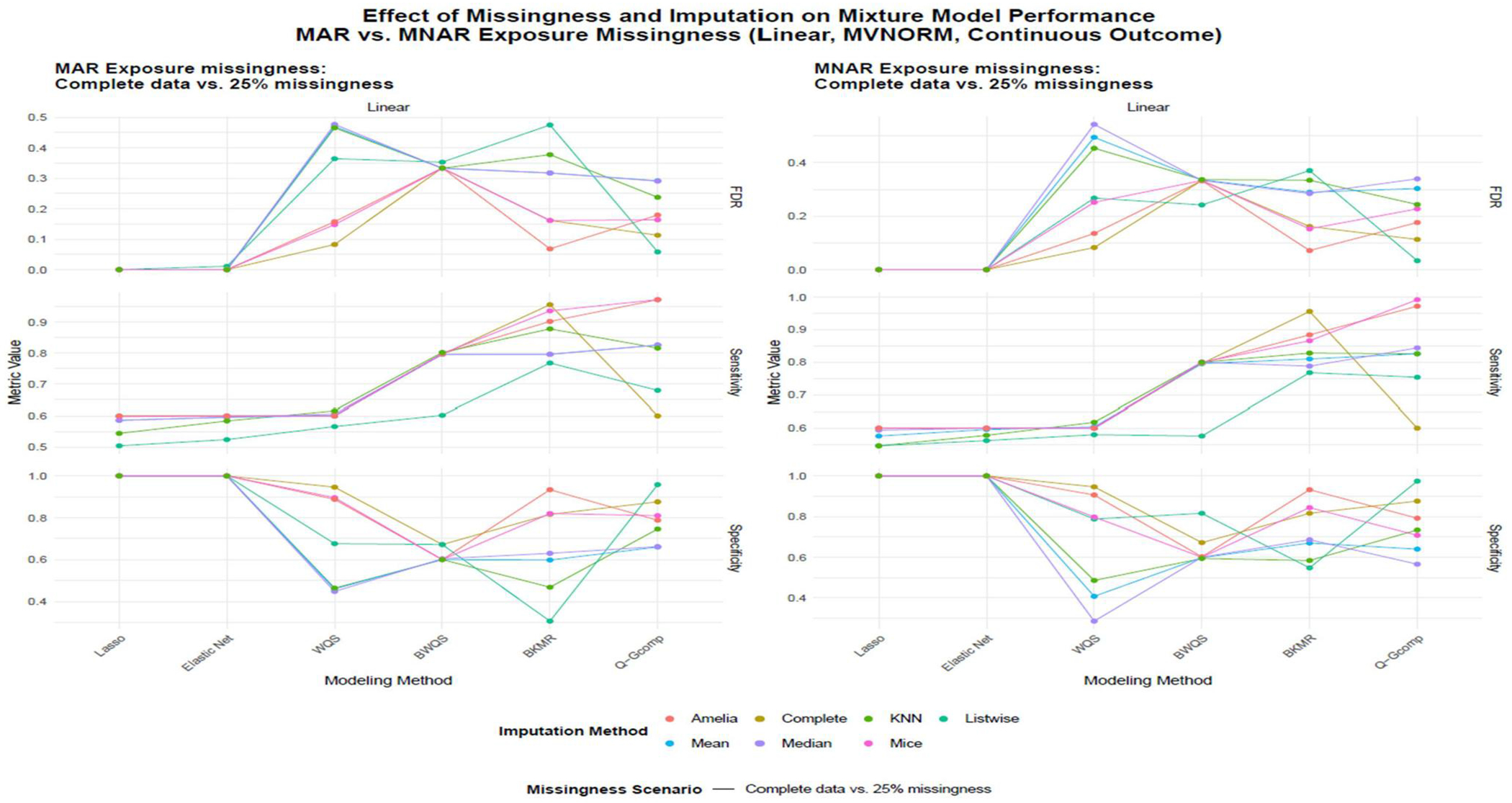
Performance of mixture modeling methods under both MAR and MNAR with multivariate normal exposures, distribution, and Continuous outcomes (Complete vs 25%).

To evaluate the impact of the missingness mechanism, we compared model performance under MAR and MNAR exposure missingness (Figure 6).

Across all models, performance deteriorated under MNAR relative to MAR, with the most pronounced effects observed in SP and FDR. Flexible models, particularly BKMR and WQS, exhibited substantial reductions in SP and corresponding increases in FDR under MNAR, indicating a greater tendency toward false positive selections when missingness depended on unobserved exposure values.

MI approaches (MICE and Amelia), which performed strongly under MAR, showed reduced stability under MNAR, with increased FDR and lower SP across several models. This decline is consistent with the MAR assumption underlying these methods.

In contrast, Q-gcomp maintained a relatively stable balance between SE, SP, and FDR across both MAR and MNAR settings, suggesting greater robustness to violations of the missing-random assumption. Penalized methods (Elastic Net and LASSO) remained conservative under both mechanisms, preserving high SP but consistently yielding lower SE.

SI methods, including mean, median, and KNN, demonstrated moderate but variable performance and did not mitigate the bias introduced under MNAR. Overall, these findings highlight that model performance is more sensitive to the missingness mechanism than to exposure distribution, particularly when the MAR assumption is violated.

### 4.5. Effect of Imputation Model Specification

**Figure 7.**
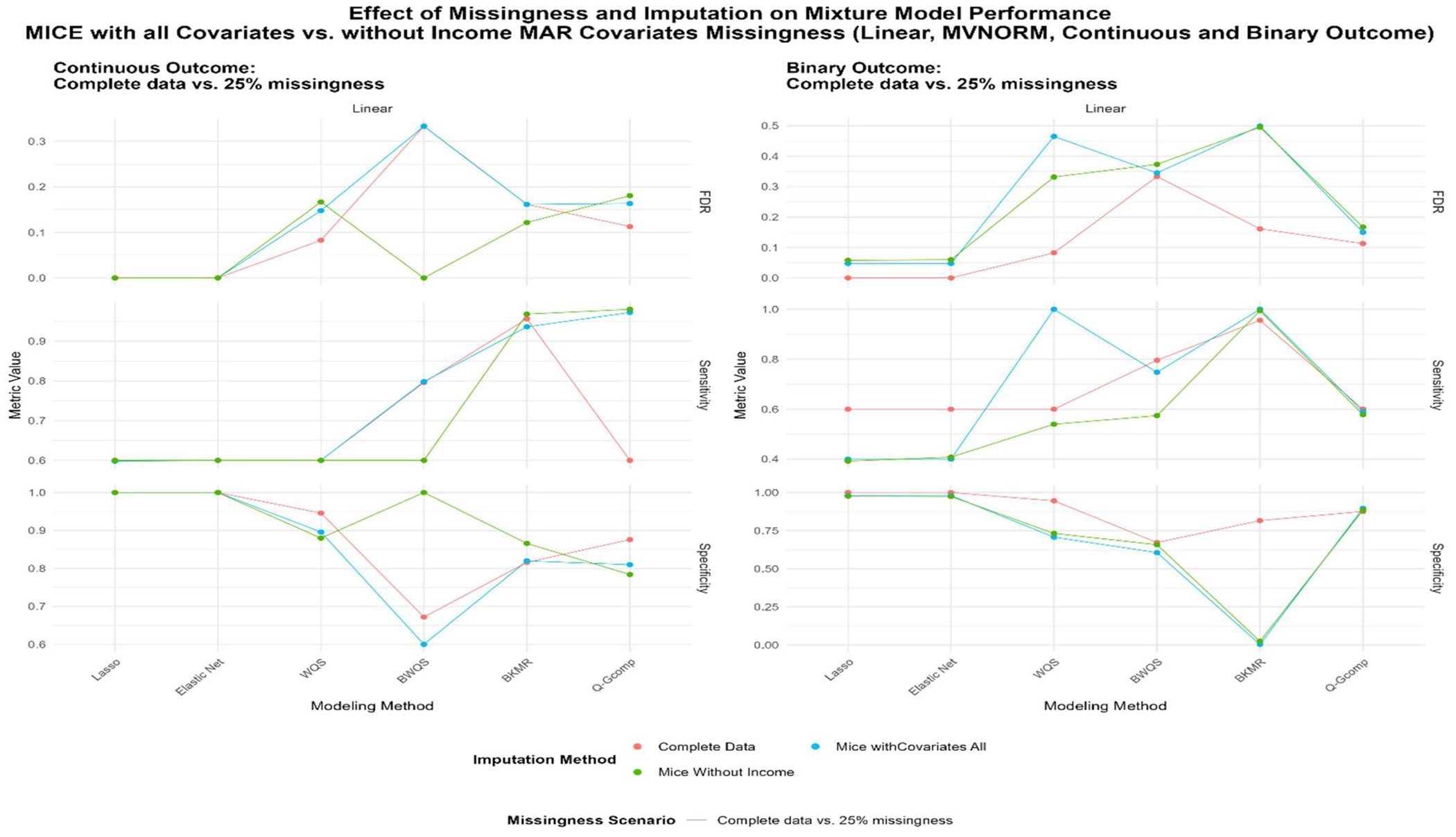
Performance of mixture modeling methods under MAR and MICE with all exposures, and without all exposures (excluding Income) with multivariate normal exposures, distribution (Complete vs 25%).

To assess the impact of imputation model specification, we compared MICE performance with and without the inclusion of an additional covariate (income) under MAR exposure missingness (Figure 7). Results indicate that inclusion of all relevant covariates in the imputation model improves performance across several metrics. Specifically, omitting income led to greater variability in SE, SP, and FDR, particularly for flexible models such as BKMR and WQS. Q-gcomp remained relatively stable, though modest declines in performance were observed when income was excluded. These effects were more pronounced for binary outcomes, in which both SE and SP showed greater variability. Overall, these findings demonstrate that imputation model specification plays a critical role in downstream mixture model performance, and omission of relevant predictors can introduce bias and reduce stability.

## 5. Application to a Study of Metal Mixtures and Systolic Blood Pressure in NHANES 2007-2014

To complement the simulation framework described above, we applied the same modeling and missing-data strategies to an empirical dataset from the National Health and Nutrition Examination Survey (NHANES) 2007–2014. NHANES is a continuous, nationally representative survey conducted by the U.S. Centers for Disease Control and Prevention (CDC) and provides comprehensive demographic, health, and environmental exposure data, making it well-suited for evaluating complex exposure–outcome relationships in real-world mixture settings. This application was designed to evaluate whether patterns observed under controlled simulation settings generalize to real-world environmental mixture data.

### 5.1. Data and Study Population

Four NHANES cycles (2007–2014) were combined to maximize sample size and ensure sufficient coverage of both PFAS and metal exposures. Participants younger than 18years, pregnant women, and those missing outcome or key covariate information were excluded. After data cleaning, harmonization, and removal of implausible values, the final analytic sample comprised 8,233 participants. This sample size and missingness structure were comparable to those used in the simulation scenarios, enabling direct comparison between simulation and empirical findings.

The merged dataset included 54 variables spanning exposure, outcome, and sociodemographic domains. Summary characteristics of the study population and exposure distributions are presented in Table 9.

To facilitate cross-method comparison, a radar (spider) chart was constructed to evaluate the six mixture-modeling approaches WQS, BWQS, Q-gcomp, BKMR, Elastic Net, and LASSO across five standardized performance dimensions (stability, cross-model agreement, interpretability, runtime efficiency, and weight dispersion).

#### 5.1.1. Outcome Measurement

The primary outcome was systolic blood pressure (SBP), measured during the NHANES mobile examination center (MEC) visit using a standardized protocol administered by trained technicians. SBP was defined as the average of three seated measurements to reduce within-subject variability and improve measurement precision.

#### 5.1.2. Covariates

Covariates were selected a priori based on their established associations with both environmental exposures and cardiovascular outcomes. These included age (years), gender (male/female), and household income (categorized as <$25,000; $25,000–$54,999; $55,000–$74,999; ≥$75,000).

Demographic and socioeconomic data were obtained from the NHANES interview and examination components and harmonized across survey cycles. NHANES design variable sample weights, strata, and primary sampling units were incorporated into descriptive analyses to ensure national representativeness, while mixture models were estimated using unweighted data to maintain comparability across modeling frameworks.

### 5.2. Handling of Missing Data

Because exposure-related missingness was nontrivial, the same missing-data strategies evaluated above were applied: listwise deletion, mean imputation, median imputation, KNN, MI by chained equations (MICE, *m* = 5), and MI *via* Amelia (*m* = 5). Imputation models included exposures, covariates, and the outcome to preserve multivariate relationships and enhance the plausibility of the missing-at-random (MAR) assumption. Results from MICE and Amelia were pooled using Rubin’s rules and served as the primary analytic strategy.

### 5.3. Statistical Analysis

The six mixture-modeling approaches were applied under each missing-data strategy All models adjusted for age, gender, and income. The primary analytic focus was variable importance and ranking stability, consistent with the evaluation framework established earlier. Variable importance was defined using model-specific metrics: posterior mean weights (BWQS), bootstrap-based weights (WQS), standardized coefficients (Q-gcomp), posterior inclusion probabilities (BKMR), and cross-validation–based selection frequencies for Elastic Net and LASSO (using *λ*_1_se).

Model results were compared across missing-data strategies to assess the reproducibility of exposure rankings, the sensitivity to missingness, and the interpretability of exposure contributions. Descriptive analyses incorporated survey design weights, whereas mixture models were estimated using unweighted data to ensure comparability across frameworks.

#### 5.3.1. Descriptive Statistics

The final analytic sample (*n* = 8,233) included four per- and polyfluoroalkyl substances (PFOA, PFOS, PFHxS, PFNA) and three metals (lead [Pb], mercury [Hg], and cadmium [Cd]) as exposures. The PFAS panel exhibited an average missingness of 24.3%, while SBP had a 4.1% missing rate. The descriptive characteristics of the study population, including sociodemographic distributions and summary statistics on exposure, are presented in Table 9.

#### 5.3.2. Variable Importance Across Mixture Models

Variable-importance estimates were derived under the MICE framework, the primary missing-data strategy, to maintain consistency with the simulation results. Overall, the empirical findings closely mirrored patterns observed in the simulation study (Section 4), with BWQS and Q-gcomp providing the most stable and interpretable profiles of variable importance. BWQS consistently prioritized PFOA and lead as the most influential exposures, whereas Q-gcomp produced directionally interpretable associations: lead was positively associated with SBP, and mercury was negatively associated. These results reinforce the complementary strengths of rank-based and regression-based mixture approaches observed in the simulation setting.

WQS produced estimates of similar magnitude but exhibited greater sensitivity to the missing-data strategy, particularly under listwise deletion, where reduced sample size inflated the apparent importance of PFOS and PFOA. BKMR posterior inclusion probabilities were uniformly low (PIP < 0.20), indicating limited evidence of dominant nonlinear or interaction-driven effects in this dataset.

**Figure 8.**
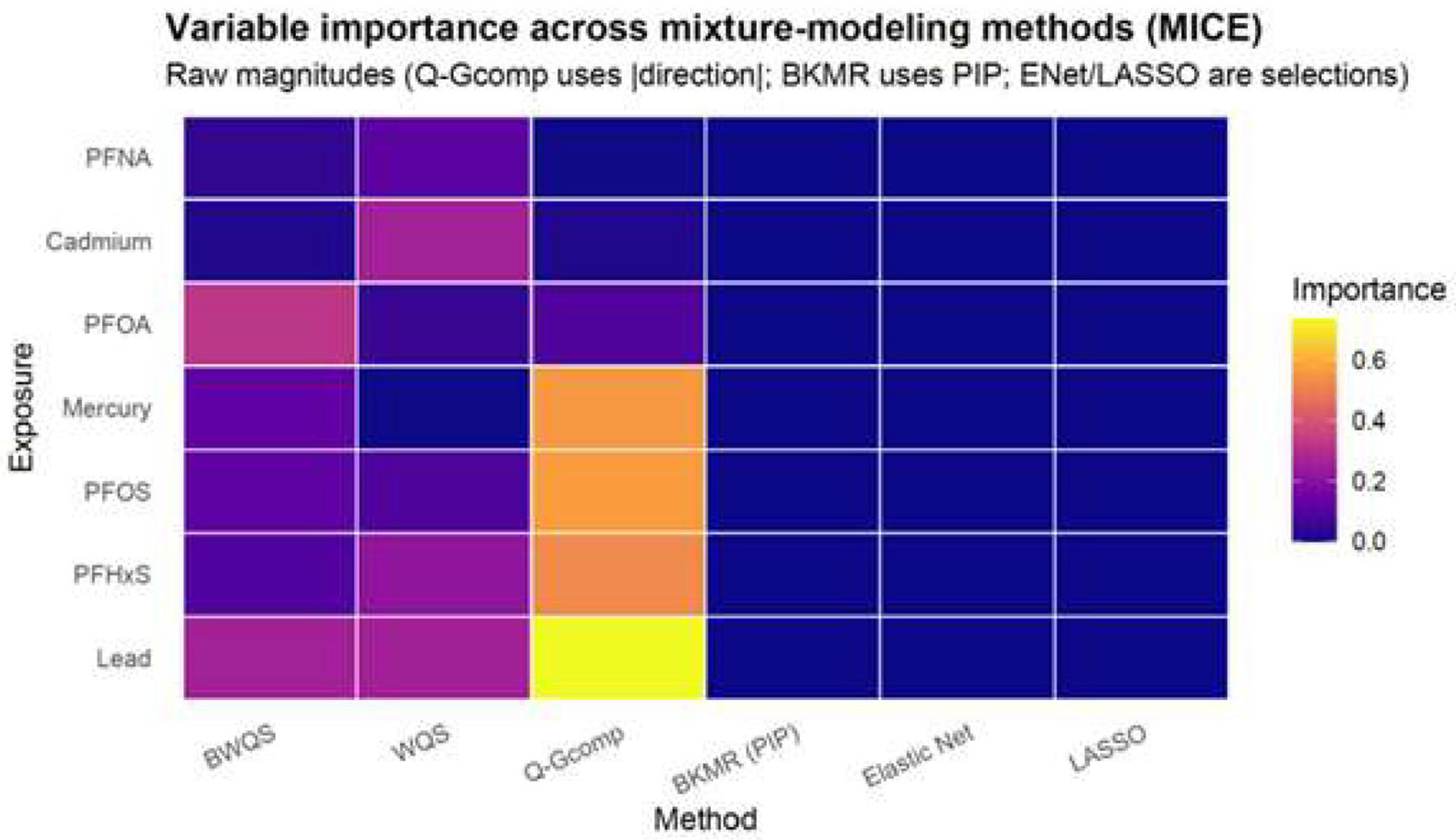
Heatmap of variable importance across mixture-modeling methods.

Penalized regression methods (Elastic Net and LASSO) retained covariates (age and gender) with nonzero coefficients, while PFAS and metal exposures were shrunk to zero. This indicates that no individual exposure exhibited a strong independent linear signal after accounting for correlation and regularization, rather than confirming a collective mixture effect. Instead, these findings suggest that exposure effects are likely distributed across the mixture and not attributable to a single dominant component. Variable-importance patterns across models are summarized in Table 10 and visualized in Figure 8, which presents a heat map of exposure contributions under multiple imputation.

#### 5.3.3. Effect of Missing-Data Strategy on Weight Stability

Consistent with the simulation finding, missing-data handling substantially influenced both the magnitude and stability of exposure weights. Listwise deletion produced the greatest variability, amplifying the apparent importance of select exposures (notably PFOS and PFOA) due to reduced sample size and information loss.

In contrast, imputation-based approaches, including KNN, MICE, and Amelia, yielded more balanced and consistent exposure rankings. While KNN improved recovery of multivariate relationships relative to simple deterministic methods, it remained more variable than MI approaches, which better accounted for uncertainty. These findings align with the simulation results, which show that MI consistently improved stability and reduced variability in model outputs.

Among mixture-modeling frameworks, BWQS and Q-gcomp demonstrated the highest weight stability, preserving the rank order of exposures across missing-data strategies. Q-gcomp consistently identified lead and PFOS among the top contributors, while BWQS prioritized lead and PFOA. In contrast, WQS exhibited greater fluctuations in weight assignments, particularly under SI (mean and median), with exposures such as PFHxS and PFNA varying in importance.

BKMR produced uniformly low PIP across all strategies, indicating minimal sensitivity to missing-data assumptions in this setting. Penalized regression models showed little variation in selection patterns across imputations, suggesting that variable inclusion was relatively insensitive to the choice of missing-data method.

Overall, MI approaches, particularly MICE and Amelia, enhanced the stability and interpretability of exposure rankings, consistent with their ability to preserve multivariate structure and account for uncertainty. KNN demonstrated intermediate performance, outperforming simple deterministic methods but not achieving the stability of MI.

**Figure 9.**
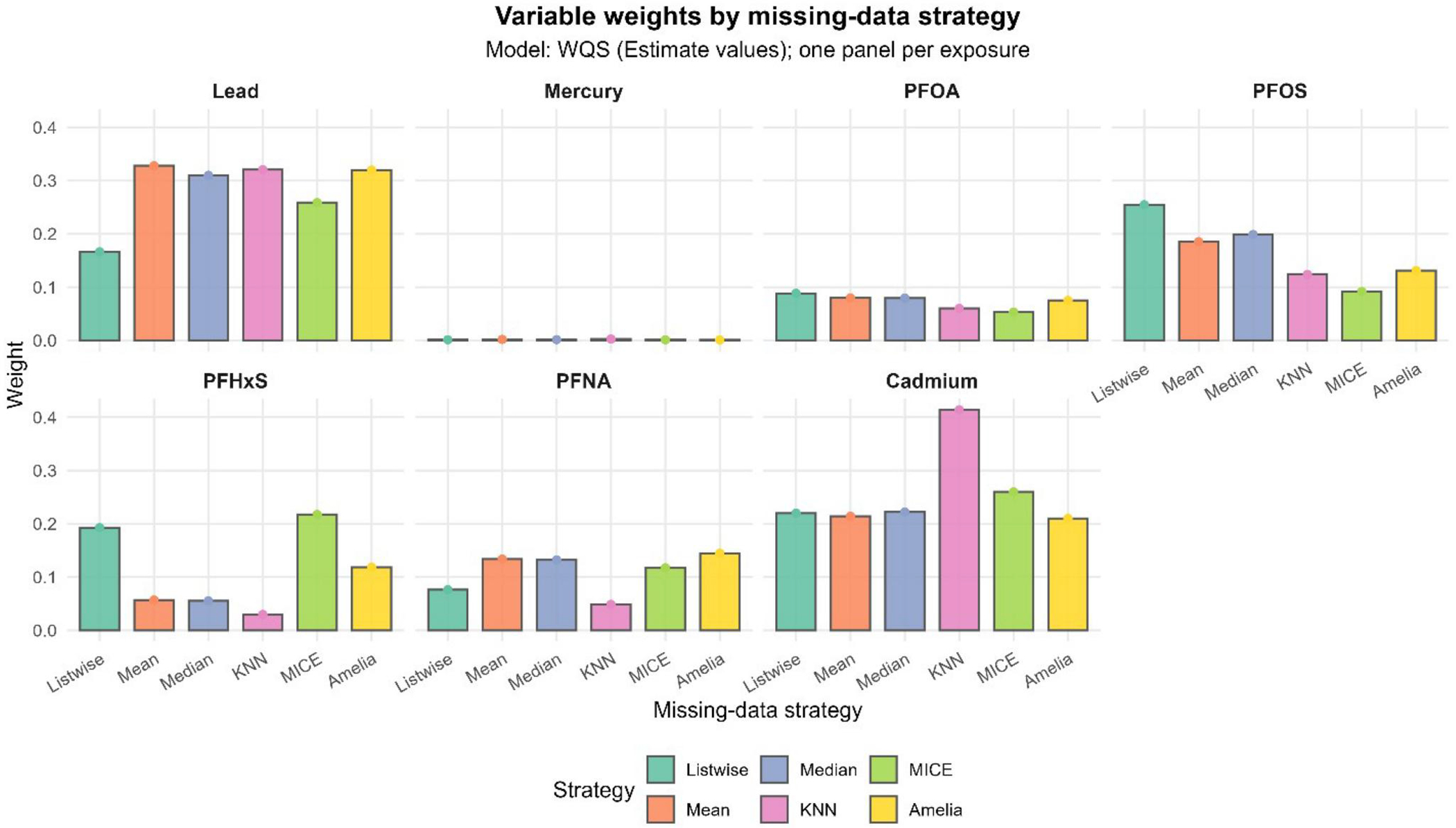
Plots of variable weights across missing data strategies for WQS.

**Figure 10.**
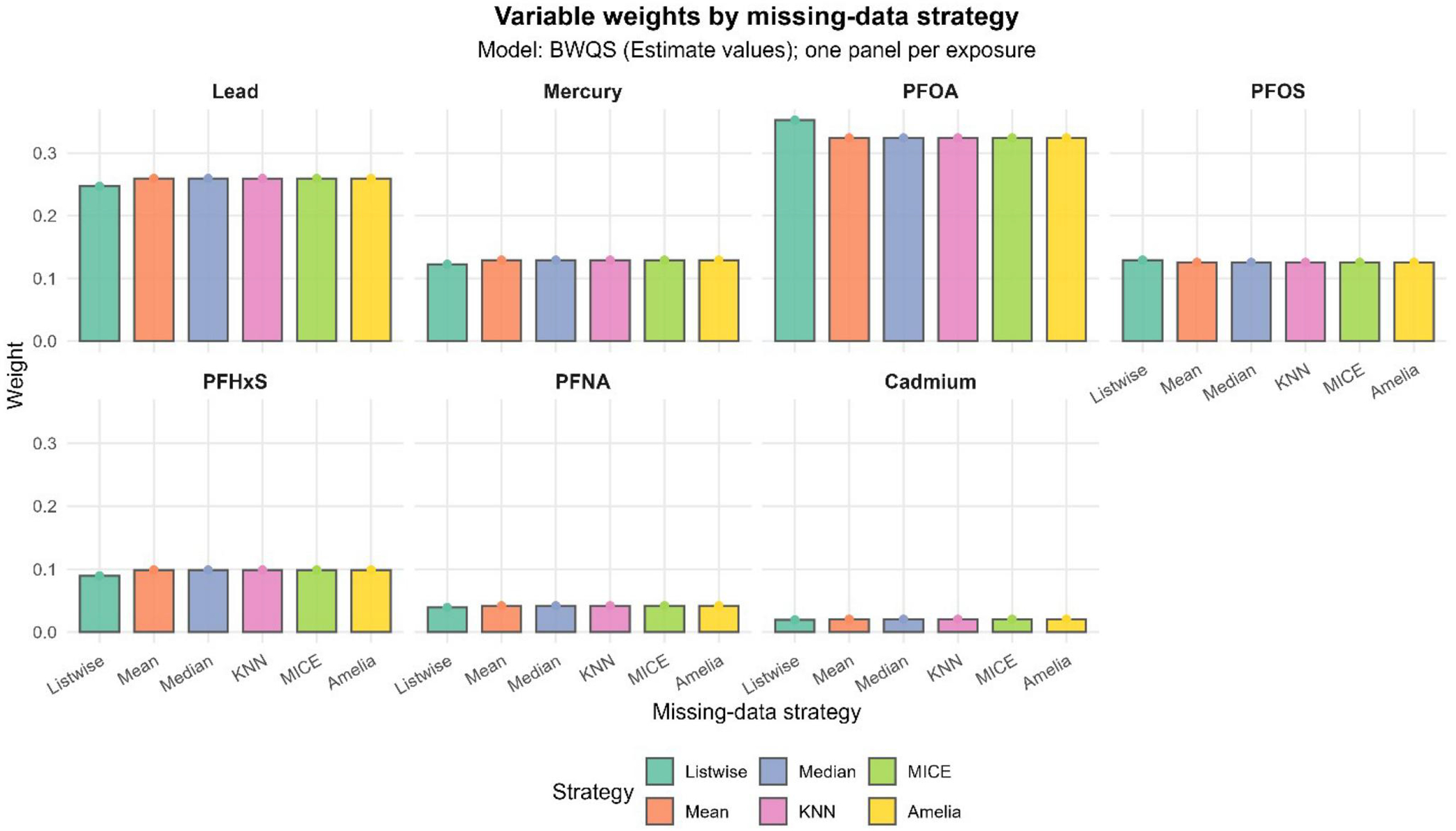
Plots of variable weights across missing data strategies for BWQS.

**Figure 11.**
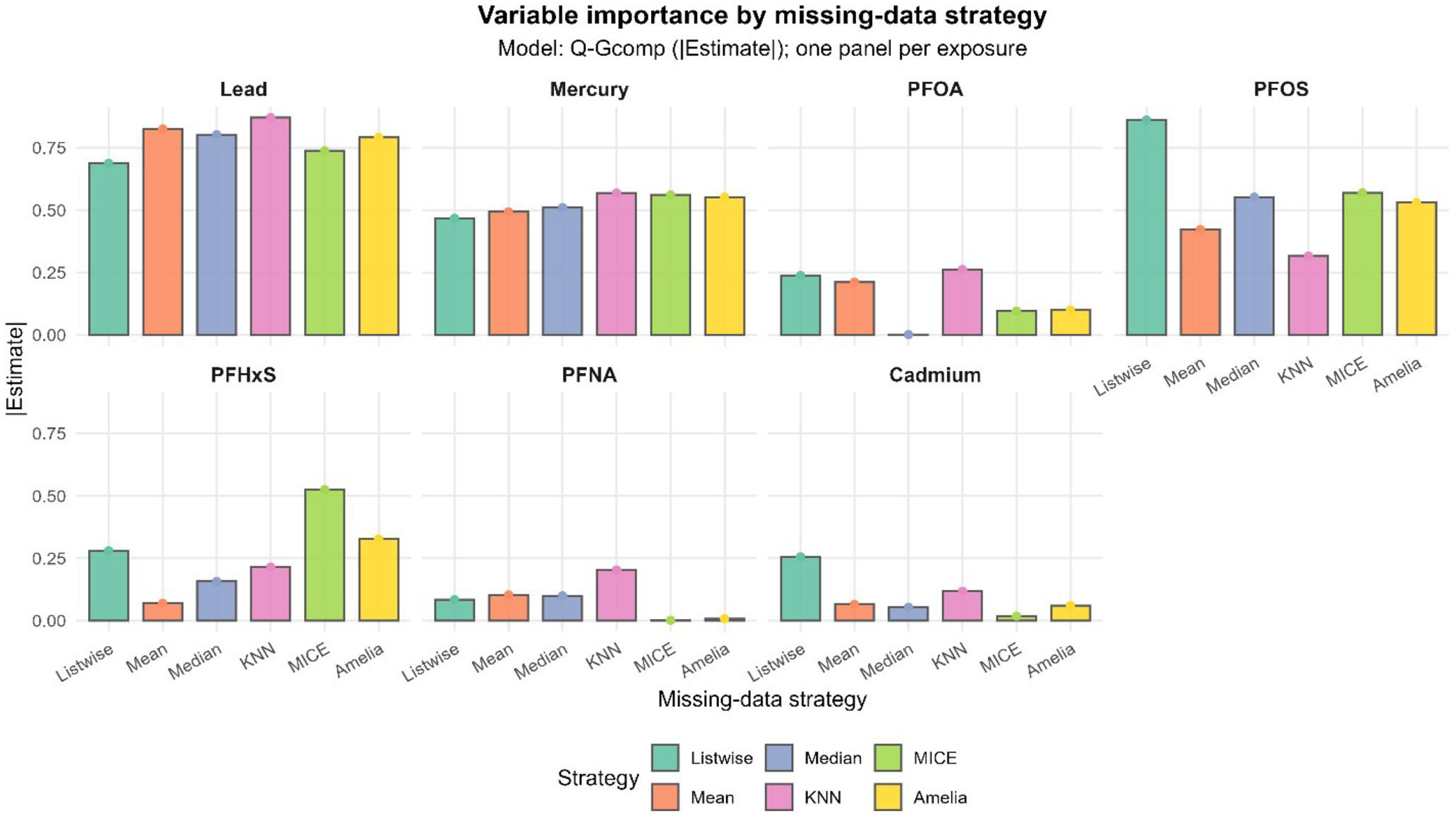
Plots of variable weights across missing data strategies for Q-gcomp.

The accompanying plots (Figures 9–11) illustrate variable-weight distributions across missing-data strategies for BWQS, WQS, and Q-gcomp, highlighting the greater consistency of Bayesian and quantile-based mixture models relative to listwise deletion and SI approaches.

#### 5.3.4. Cross-Method Rank Consistency and Stability Metrics

To assess cross-model agreement, pairwise Spearman correlations were computed between exposure-importance rankings derived from the six modeling frameworks under the MICE imputation scenario. Overall, patterns of agreement were modest but consistent with those observed in the simulation study, reflecting differences in model structure and underlying assumptions.

BWQS and Q-gcomp exhibited the highest agreement (*ρ* = 0.36), followed by WQS and Q-gcomp (*ρ* = 0.29). In contrast, BWQS and WQS showed an inverse relationship (*ρ* = −0.54), suggesting that the directional constraint imposed by WQS may alter rank ordering relative to the unconstrained Bayesian and quantile-based approaches. These results highlight that, while methods may target similar inferential goals, differences in modeling assumptions can lead to variation in exposure rankings.

BKMR produced uniformly low PIPs (PIPs ≈ 0), limiting its ability to generate meaningful rank differentiation and leading to undefined cross-model correlations. Similarly, Elastic Net and LASSO did not retain exposures with non-zero coefficients, yielding undefined rank correlations, reinforcing the idea that penalized regression primarily serves as a screening tool for identifying dominant individual predictors rather than ranking distributed mixture effects.

Stability was assessed using the coefficient of variation of exposure weights across imputations. BWQS (0.09) and Q-gcomp (0.12) exhibited the lowest variability, indicating the highest reproducibility of exposure rankings and the least sensitivity to imputation uncertainty. These findings are consistent with the simulation results, which show that both methods demonstrate strong stability under imputation. Cross-method agreement and stability metrics are summarized in Table 11.

**Figure 12.**
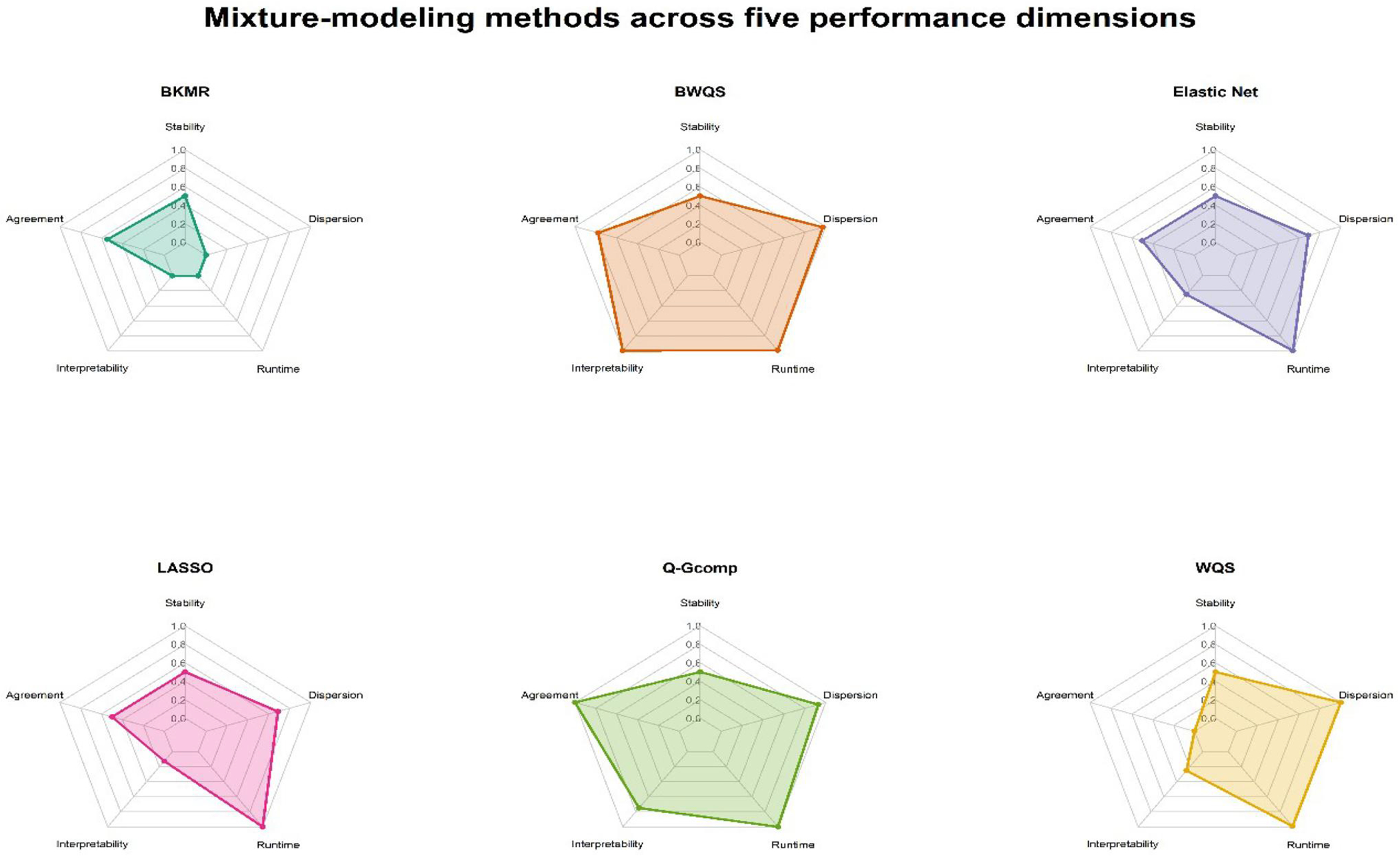
Radar chart comparing mixture-modeling methods across five performance dimensions.

The radar plot (Figure 12) summarizes model performance across five standardized dimensions: stability, cross-model agreement, interpretability, runtime efficiency, and weight dispersion. Larger, more balanced polygons indicate stronger, more consistent overall performance. BWQS and Q-gcomp exhibit the most consistent profiles, reflecting high SE, controlled FDR, and strong agreement across methods. WQS demonstrates moderate performance with lower agreement and greater sensitivity in the imputation strategy. BKMR shows reduced stability and higher computational burden, while Elastic Net and LASSO offer strong runtime efficiency but reduced interpretability due to coefficient shrinkage and less stable variable selection.

Overall, these results indicate that Bayesian and quantile-based approaches provide the most stable and interpretable assessments of variable importance in environmental mixture analyses, particularly under multiple-imputation settings.

#### 5.3.5. Model-Specific Observations

Overall, model behavior in the NHANES application was consistent with patterns observed in the simulation study. BWQS demonstrated the highest stability and interpretability of exposure importance across missing-data strategies, consistently identifying key contributors such as lead and PFOA. Q-gcomp provided directionally interpretable coefficients with moderate consistency in ranking, reinforcing its utility for assessing both magnitude and direction of associations.

WQS produced broadly similar rankings but was more sensitive to missing-data handling, particularly under listwise deletion, where reduced sample size led to inflated weights for select exposures. BKMR yielded uniformly low posterior inclusion probabilities, suggesting limited evidence of dominant nonlinear or interaction-driven effects in this dataset.

Penalized regression methods (Elastic Net and LASSO) retained only a small subset of variables, primarily covariates, while shrinking exposure coefficients toward zero. This indicates that no individual exposure exhibited a strong independent linear signal after accounting for correlation within the mixture, rather than implying the absence of association.

#### 5.3.6. Comparative Model Performance and Recommended Applications

Taken together, these results highlight the complementary strengths of the evaluated mixture-modeling approaches, consistent with the simulation findings. BWQS excelled at producing stable, interpretable exposure rankings, while Q-gcomp provided clear insight into the direction and magnitude of associations. WQS remains useful as a sensitivity or screening tool but is more susceptible to instability under incomplete data.

BKMR offers flexibility for detecting nonlinear and interaction effects but demonstrated limited discriminatory power in this application and a higher computational burden. In contrast, Elastic Net and LASSO provide efficient variable selection and strong control of false positives but may fail to detect distributed effects across highly correlated exposures.

The tendency of penalized models to shrink exposure coefficients toward zero underscores a key limitation in mixture settings: these approaches are designed to identify dominant individual predictors and may not capture cumulative or joint effects. As a result, mixture-specific methods such as BWQS and Q-gcomp are better suited for characterizing distributed exposure effects in correlated environmental mixtures.

A summary of comparative model performance and recommended applications is provided in Table 12, which synthesizes findings across simulation and empirical analyses.

## 6. Discussion

Environmental mixture analyses are inherently challenging due to correlated exposures, potential nonlinear relationships, and the presence of missing data, all of which can bias inference if not properly addressed (Van Buuren and Groothuis-Oudshoorn 2011; Carrico et al. 2015; Hao et al. 2024). By combining simulation and empirical analyses, this study provides a comprehensive evaluation of how imputation strategies and modeling approaches jointly influence inference in environmental mixture settings.

Across both MVNORM and MVT scenarios, MI methods, particularly MICE and Amelia, consistently outperformed simple deterministic approaches by preserving key statistical properties and maintaining stable performance across SE, SP, and FDR. In contrast, SI methods underestimated variability and reduced inferential reliability, consistent with prior findings (Eekhout et al. 2014; Gômez-Carracedo et al. 2014). KNN demonstrated intermediate performance, improving upon simple methods but failing to capture uncertainty to the same extent as MI. Together, these results reinforce the importance of accounting for imputation uncertainty in mixture analyses, particularly when distributions are non-normal.

Model performance varied according to both data structure and analytic objective. BKMR and Q-gcomp demonstrated the most consistent overall performance across simulation scenarios, with BKMR effectively capturing nonlinear and interaction effects (Bobb et al. 2015). Q-gcomp provides robust estimates under linear structures. In contrast, Elastic Net and LASSO offered strong SP and control of false positives but at the cost of reduced SE, reflecting their emphasis on identifying dominant individual predictors. WQS and BWQS showed greater sensitivity under distributional assumptions, particularly in MVT settings, where inflated FDRs were observed, consistent with previous evaluations.

Missingness played a critical role in shaping model performance. Combined exposure–outcome missingness posed the greatest challenge, leading to declines in both SE and SP. However, MI strategies substantially mitigated these effects, whereas simpler methods failed to recover the underlying signal. These findings highlight that the impact of missing data are not uniform but interacts with both the underlying data distribution and the modeling framework, underscoring the need for robust imputation strategies in environmental mixture research.

### 6.1. Runtime and Efficiency

Computational burden was strongly influenced by the choice of missing-data strategy. Simple single-value imputations (mean and median) ran near baseline, were computationally efficient, but provided limited statistical reliability. KNN imputation introduced a moderate increase in computational cost due to the need to compute distances across observations. While it improved recovery of multivariate structure compared to simple deterministic methods, it remained less efficient than simpler approaches and did not account for imputation uncertainty. MI approaches, particularly MICE and Amelia, required additional computational effort due to iterative estimation and repeated model fitting across imputations. However, these methods provided more stable and reliable results, and their computational cost was manageable relative to the gains in statistical rigor.

Model-specific differences further influenced runtime. BKMR consistently exhibited the highest computational burden, driven by its Bayesian sampling and nonlinear modeling. In contrast, Q-gcomp, Elastic Net, and LASSO were computationally efficient across all settings. WQS and BWQS demonstrated moderate runtime, though they showed some sensitivity to the underlying imputation method.

Overall, these findings highlight a tradeoff between computational efficiency and inferential quality. While simpler imputation methods offer speed, they may compromise reliability. KNN provides an intermediate balance between structural recovery and computational cost but does not account for uncertainty. MI approaches, despite increased computational demands, offer the most robust and reproducible results and are therefore preferable when missingness is nontrivial or data complexity is high.

### 6.2. Practical Guidance

Table 13 summarizes the recommended modeling and imputation strategies based on findings from both the simulation and NHANES application analyses. For linear continuous outcomes, Q-gcomp combined with MI provides strong SE and interpretability, whereas for nonlinear continuous outcomes, BKMR with MI performs reliably across both MVNORM and MVT settings. Listwise deletion is generally not recommended and should be limited to scenarios where missingness is minimal and plausibly missing completely at random (MCAR). For binary outcomes, Q-gcomp provides stable control of FDR and SP, whereas penalized regression methods are preferable when minimizing false positives is the primary objective. WQS and BWQS, when used as exploratory indices, should be interpreted with caution, particularly under heavy-tailed (MVT) distributions.

Findings from the NHANES application reinforce that conclusions regarding variable importance depend jointly on the modeling framework and the handling of missing data. BWQS and Q-gcomp produced the most stable and interpretable exposure rankings across strategies, whereas WQS exhibited greater sensitivity to missing-data handling, particularly under listwise deletion, which amplified the apparent influence of select exposures (notably PFOS and PFOA) as sample size decreased. In contrast, MI approaches, especially MICE and Amelia, yielded more balanced and reproducible results, reflecting their ability to preserve multivariate structure and propagate imputation uncertainty (Rubin 1976; Honaker et al. 2011; Van Buuren 2018). KNN demonstrated intermediate performance, improving upon simple deterministic methods but not achieving the stability of multiple imputations.

Across models, the convergence of BWQS and Q-gcomp on similar top-ranked contributors under MI suggests that rank-based (BWQS) and marginal-contrast (Q-gcomp) frameworks can provide broadly concordant evidence on component importance when correlations are moderate and missingness is appropriately addressed. This observation is consistent with prior work demonstrating improved weight stability in Bayesian WQS models (Colicino et al. 2020) and the interpretability of Q-gcomp coefficients under correlated exposures (Keil and Keil 2020). At the same time, moderate cross-method agreement highlights that differences in model assumptions can lead to variation in exposure rankings.

BKMR produced uniformly low PIPs, indicating limited evidence for dominant nonlinear or interaction effects in this dataset. This may reflect either distributed effects across the mixture or reduced power to detect nonlinear dominance under missingness and variability, suggesting that BKMR can serve as a useful diagnostic tool when strong signals are absent.

Penalized regression methods (Elastic Net and LASSO) provided complementary insight. Under cross-validated tuning, these models did not retain PFAS or metal exposures with nonzero coefficients, while covariates (e.g. age and gender) remained selected. This pattern is consistent with scenarios in which correlated exposures exert modest, distributed effects that are difficult to isolate using sparsityinducing methods (Tibshirani 1996; Zou and Hastie 2005). Rather than indicating the absence of association, these findings support a mixture-driven interpretation, where cumulative effects across exposures are more relevant than any single dominant component.

Methodologically, these findings support three key recommendations for environmental mixture analyses. First, MI should be prioritized over listwise deletion when missingness is nontrivial, as MI reduces between-imputation variability, improves rank stability, and preserves multivariate relationships, whereas listwise deletion can introduce bias due to information loss and violations of the MAR assumption (Rubin 1976; Van Buuren 2018). Second, model selection should be aligned with the analytic objective: BWQS is well-suited for estimating relative importance with uncertainty quantification, Q-gcomp provides interpretable estimates of direction and magnitude, BKMR is appropriate when nonlinear or interaction effects are of interest, and Elastic Net/LASSO are useful for testing whether any single exposure persists after penalization. Third, cross-method comparison should be incorporated into mixture analyses. Reporting rank correlations and stability metrics helps distinguish robust signals from method-specific artifacts and enhances reproducibility.

Overall, these results demonstrate that model choice should be guided by the analytic objective rather than reliance on a single method or fit statistic. BWQS and Q- gcomp provide complementary perspectives on variable importance and effect direction, and their joint use within a MI framework enhances interpretability, stability, and transparency in environmental mixture analyses.

### 6.3. Limitations and Implications

Several limitations should be considered. While the simulation framework incorporated both MAR and MNAR mechanisms, the empirical application relied on MAR-based imputation methods, which may not fully capture the complexity of real-world missingness. Future work should extend these analyses to MNAR settings and develop methods robust to violations of MAR assumptions.

Additionally, although widely used mixture-modeling approaches were evaluated, the inclusion of additional methods could further broaden the comparative framework. Finally, validation in larger datasets and under more complex exposure–outcome relationships would improve generalizability.

Despite these limitations, this study provides a comprehensive evaluation of how imputation strategies and modeling approaches jointly influence inference in environmental mixture analyses. The integration of simulation and real-world data strengthens the robustness and applicability of the findings.

## 7. Conclusion

In conclusion, this study demonstrates the critical role of missing-data handling in environmental mixture modeling and provides evidence-based guidance for selecting appropriate analytical strategies. MI methods, particularly MICE and Amelia, consistently improved stability and inferential reliability across both simulated and empirical settings. By integrating robust imputation techniques with complementary modeling approaches such as BKMR and Q-gcomp, researchers can better capture complex mixture effects while maintaining interpretability and reproducibility. These findings advance methodological rigor in environmental health research and support more reliable identification of exposure–outcome relationships in the presence of missing data.

## Figures and Tables

**Table 1.** Model performance summary by distribution.

Model	Normal Distribution	MVT Distribution
Lasso	High specificity, low sensitivity, low FDR.	High specificity, low sensitivity.
Elastic Net	High specificity, low sensitivity, low FDR.	High specificity, slightly lower sensitivity.
WQS	Performance varies by imputation method.	Sensitivity improves under MVT.
BWQS	Moderate sensitivity and specificity.	Improved sensitivity under MVT.
BKMR	Varies by imputation; strongest with MI.	High sensitivity with reduced specificity.
Q-gcomp	Strong balance across metrics.	Strong balance across metrics.

**Table 2.** Missing data performance summary by distribution.

Method	Normal Distribution	MVT Distribution
Listwise Deletion	Reasonable specificity and FDR control.	Maintains specificity with slight sensitivity gains.
Mean Imputation	Does not preserve multivariate relationships.	Slight nonlinear improvement.
Median Imputation	Fails to preserve relationships.	Remains ineffective.
KNN Imputation	Moderate; improved over mean/median but variable.	Moderate with slight sensitivity gain.
MICE	Preserves sensitivity and FDR.	Improves sensitivity and FDR.
Amelia	Best overall preservation.	Strong across settings.

**Table 3.** Model performance summary by relationship.

Model	Linear Association	Non-Linear Association
Lasso	High specificity, low sensitivity, low FDR.	Similar pattern.
Elastic Net	Similar to Lasso.	Slight improvement.
WQS	Varies by imputation.	Slight sensitivity gain.
BWQS	Moderate performance.	Slight improvement.
BKMR	Varies; poor under listwise.	Strong under nonlinear.
Q-gcomp	Strong balance.	Best balance.

**Table 4.** Missing data performance summary by relationship (continuous outcome).

Method	Linear	Non-Linear
Listwise	Maintains specificity for penalized models.	Maintains specificity.
Mean	Low FDR, reduced sensitivity.	Balanced.
Median	Similar to mean.	Similar.
KNN	Moderate improvement over mean/median.	Moderate with variability.
MICE	Improves sensitivity.	Improves sensitivity.
Amelia	Similar to MICE.	Balanced.

**Table 5.** Missing data performance summary by relationship (binary outcome).

Method	Linear	Non-Linear
Listwise	Preserves specificity/FDR.	High specificity.
Mean	Low FDR, reduced sensitivity.	Balanced.
Median	Similar to mean.	Similar.
KNN	Improved sensitivity, variable specificity.	Moderate.
MICE	Improves sensitivity.	Improves sensitivity.
Amelia	Balanced improvement.	Balanced.

**Table 6.** Missing data performance summary by distribution (binary outcome).

Method	MVNORM	MVT
Listwise	Reasonable specificity.	Slight sensitivity gain.
Mean	Reduced sensitivity.	Slight improvement.
Median	Limited.	Limited.
KNN	Moderate.	Moderate improvement.
MICE	Improves sensitivity.	Improves performance.
Amelia	Best overall.	Strong.

**Table 7.** Model performance summary by relationship (binary outcome).

Model	Linear	Non-Linear
Lasso	High specificity, low sensitivity.	Moderate sensitivity.
Elastic Net	Similar to Lasso.	Most balanced penalized.
WQS	High sensitivity, high FDR.	Same pattern.
BWQS	Moderate.	Slight improvement.
BKMR	Very high sensitivity, low specificity.	Same.
Q-gcomp	Best balance.	Best.

**Table 8.** Model performance summary by distribution (binary outcome).

Model	MVNORM	MVT
Lasso	High specificity, low sensitivity.	Slightly lower sensitivity.
Elastic Net	High specificity, low sensitivity.	Improved sensitivity.
WQS	High sensitivity, high FDR.	Same.
BWQS	Moderate.	Improved sensitivity.
BKMR	Over-selects.	Extreme sensitivity.
Q-gcomp	Strong balance.	Strong balance.

Note. SE = Sensitivity; SP = Specificity; FDR = False Discovery Rate; MI = Multiple Imputation (MICE/Amelia).

**Table 9.** Descriptive characteristics and missingness in analytic NHANES sample (*n* = 8,233).

Variable	Mean ± SD or %	Missing %
Age (years)	43.07 ± SD 18.77	0
Male (%)	51.11 %	0
Income (<$ 25,000)	32.0%	0
Income ($ 25,000–$54 999)	42.3%	0
Income ($ 55,000–$74 999)	20.1%	0
Income (≥$75,000)	5.6%	0
PFOA (ng/mL)	3.70 ± SD 2.85	24.35%
PFOS (ng/mL)	12.82 ± SD 13.06	24.35%
PFHxS (ng/mL)	2.50 ± SD 3.34	24.35%
PFNA (ng/mL)	1.39 ± SD 1.36	24.35%
Lead (μg/dL)	1.48 ± SD 1.70	0
Mercury (μg/L)	1.50 ± SD 2.47	0
Cadmium (μg/L)	4.49 ± SD 0.60	0
SBP (mmHg)	119.11± SD 16.66	4.10%

**Table 10.** Variable importance across mixture-modeling frameworks under multiple imputations (MICE).

Exposure	BWQS Weight	WQS Weight	Q-gcomp	BKMR PIP	Elastic Net / LASSO Selection (%)
Lead	0.2595	0.2587	0.7378	[0]	[.]
Mercury	0.1291	0.0012	−0.5611	0.152	[.]
PFOA	0.3242	0.0532	0.0957	[0]	[.]
PFOS	0.1256	0.0918	0.5694	[0]	[.]
PFHxS	0.0991	0.2175	0.5251	[0]	[.]
PFNA	0.0420	0.1176	−0.0015	[0]	[.]
Cadmium	0.0205	0.2599	−0.0182	[0]	[.]

**Table 11.** Cross-method agreement and stability metrics for variable-importance ranks.

Model Pair	Spearman *ρ*	Mean SD of Weights	Stability Index	Notes
BWQS vs Q-gcomp	0.36	0.032 ± 0.004	0.09	Highest concordance under MICE
BWQS vs WQS	−0.54	0.041 ± 0.006	0.11	Inverse ordering due to WQS directionality
WQS vs Q-gcomp	0.29	0.045 ± 0.005	0.13	Low–moderate agreement
BKMR vs BWQS	NA	0.058 ± 0.010	0.18	PIPs ≈ 0; no rank variation
Elastic Net vs LASSO	NA	0.052 ± 0.008	0.15	All coefficients = 0

**Table 12.** Recommended model selection by analytic goal.

Analytic Goal	Primary Model	Preferred Missing-Data Strategy	Key Outputs
Variable importance (relative contribution)	BWQS	MICE or Amelia	Posterior weights and ranks
Direction and magnitude (component-level)	Q-gcomp	MICE	Standardized coefficients
Sensitivity to missingness	WQS	Listwise and MICE	Weight shifts across imputations
Nonlinearity and dominance	BKMR	MICE	Posterior inclusion probabilities
Linear independence check	Elastic Net/LASSO	MICE	Selected exposures and penalized coefficients

**Table 13.** Recommended methods under MAR, across outcomes and distributions.

Goal	Continuous Outcomes	Binary Outcomes
Best overall balance	Q-gcomp (all imputations; stable under MVNORM/MVT)	Q-gcomp (simple imputation or listwise; MICE/Amelia for higher sensitivity)
High sensitivity	BKMR with MICE/Amelia (robust across MVNORM/MVT)	Q-gcomp with MICE/Amelia
Low FDR / precision focus	Penalized regression (Lasso/ENet)	Q-gcomp with mean/median or listwise (5–10%)
Distributional robustness	Q-gcomp and BKMR (stable across MVNORM/MVT)	Q-gcomp (stable across MVNORM/MVT)
Use with Caution	WQS/BWQS under MVT (inflated FDR)	BKMR (low specificity), WQS/BWQS (inflated FDR under MVT)

## Data Availability

NHANES data used in this study are publicly available through the National Center for Health Statistics: https://wwwn.cdc.gov/nchs/nhanes/. The simulation and analysis code supporting this study are publicly available on GitHub: https://github.com/ysboafo-coder/Evaluating-Missing-Data-Imputation-Strategies-for-Environmental-Mixture-Models-An-Empirical-Study
